# Supplementary material for: The highly rugged yet navigable regulatory landscape of the bacterial transcription factor TetR
Source: Nat Commun. 2024 Dec 30;15:10745. doi: 10.1038/s41467-024-54723-y (PMC11686294; doi:10.1038/s41467-024-54723-y)
Supplement: Supplementary file 1 — Supplementary Information [file 41467_2024_54723_MOESM1_ESM.pdf]

The highly rugged yet navigable regulatory landscape of the bacterial transcription factor TetR

Authors

Cauã Antunes Westmann[1,2], Leander Goldbach[1,2], Andreas Wagner[1,2,3*]

[1] – Department of Evolutionary Biology and Environmental Studies, University of Zurich, Winterthurerstrasse 190, Zurich CH-8057, Switzerland

[2] – Swiss Institute of Bioinformatics, Quartier Sorge-Batiment Genopode, 1015 Lausanne, Switzerland

[3] – The Santa Fe Institute, Santa Fe, NM 87501, USA

* - Corresponding author: andreas.wagner@ieu.uzh.ch

Supplementary Information

Index

Supplementary Tables

S1. Network metrics
S2. Table of strains
S3. Table of plasmids
S4. Primers for engineering the pCAW-sort-seq plasmid
S5. Primers for constructing libraries
S6. Primers with barcodes for sequencing bins

Supplementary Figures

S1. The pCAW-SortSeq-TetR plasmid.
S2. The repression measurement module of the pCAW-SortSeq-TetR plasmid
S3 Validating the pCAW-SortSeq-TetR plasmid with *tetO2* mutants
S4. Flow cytometry gating and fluorescence expression levels for controls and library
S5. Distribution of fluorescence levels after sorting of cells with the library into bins.
S6. Distribution of sequence reads per bins
S7. Number of genotypes obtained by choosing different sequencing depth (read count) cutoffs
S8. Number of bins per genotype
S9. Correlation of read coverage in the *tetO2* mutant library
S10. Genotypes per fluorescence bin and overlaps between them
S11. Reproducibility of estimated repression levels
S12. Validation of regulatory strength differences in the lowest bins
S13. DNA Sequence LOGO obtained by a previous study
S14. Quantitative analysis of TetR landscape sparsity
S15. The contribution of individual *tetO2* nucleotides in principal component analysis
S16. Principal component analysis (PCA) of repression strength peaks in genotype space
S17. The distribution of basin sizes among peaks
S18. Adaptive walks in which each mutational step is chosen with uniform probability among all repression-increasing steps
S19. Adaptive walks using the Kimura model with different population sizes
S20. Greedy adaptive walk simulations
S21. The effect of distinct experimental noise values (τ) on landscape features
S22. The effect of experimental measurement noise on high peak attainability.
S23. Experimental noise values have little impact on genotype visitation during adaptive walks
S24. Epistatic interactions

Supplementary Tables

Table S1. Network metrics

Number of nodes (genotypes)	17,765
Number of peaks	2,092
Number of low peaks[1]	2,034 (97.2%)
Number of high peaks[2]	58 (2.8%)
Number of squares[3]	83,100
Magnitude epistasis or additivity[4,5]	35%
Simple sign epistasis[5]	34%
Reciprocal sign epistasis[5]	30%

[1] Low peaks are peaks with repression levels below the wild sequence. Percentages refer to the proportion of all genotpyes.
[2] High peaks are peaks with repression levels above the wild-type sequence. Percentages refer to the proportion of all peaks.
[3] A square represents the connection between a focal sequence (ab) and a double mutant (AB) via two single mutants (Ab and aB).
[4] This category includes both magnitude epistasis and additivity (no epistasis) without distinguishing them, because neither of the two subcategories affects peak accessibility [1,2]
[5] Percentages refer to the proportion of all squares.

Table S2. Table of strains

Strain	Genotype
SIG10-MAX from Sigma Aldrich	F- mcrA Δ(mrr-hsdRMS-mcrBC) endA1 recA1 Φ80dlacZΔM15 ΔlacX74 araD139 Δ(ara,leu)7697galU galK rpsL nupG λ- tonA (StrR)

Table S3. Table of plasmids used in this study

Name	Selective antibiotics (concentration µg/ml)	Relevant features	Source	T, °C	Description
**pCAW-Sort-Seq**	Chloramphenicol (50)	pBBR1, TetR, *sfgfp*	This study	37	Vector used for library generation and sort-seq
**pCAW-Sort-Seq-Neg**	Chloramphenicol (50)	pBBR1, TetR, promoterless *sfgfp*	This study	37	pCAW-Sort-Seq vector without a promoter for *sfgp* (negative control)

Table S4. Primers for engineering the pCAW-sort-seq plasmid

Name	Sequence	Function
pCAW_frag1_F	CGTCCGACTTACGGAAGGTAGATTTTACGGC	Linearizing the pCAW-Sort-Seq fragment1 for Gibson Assembly
pCAW_frag1_R	CTCGTGCCTAACGGAAGGTAGATTTTACGGC	Linearizing the pCAW-Sort-Seq fragment1 for Gibson Assembly
pCAW_frag2_F	TAAGATTGCCACGGAAGGTAGATTTTACGGC	Linearizing the pCAW-Sort-Seq fragment2 for Gibson Assembly
pCAW_frag2_R	AGGCCTGACTACGGAAGGTAGATTTTACGGC	Linearizing the pCAW-Sort-Seq fragment2 for Gibson Assembly

Table S5 Primers for constructing libraries

Name	Sequence	Function
Ultramer_ds_F	TTCTCAAAAGCTTCCTGCAGTATTC	Amplifying Ultramer® libraries
Ultramer_ds_R	CGGAAAGCACATCCGGTGAC	Amplifying Ultramer® libraries
TFBS_R	CCGTTTGTAGCATCACCTTC	Sequencing the TFBS region
pCAW_Gibs_Lib_F	GTCTGATGAGTCCGTGAGGACG	Linearizing the pCAW-Sort-Seq plasmid
pCAW_Gibs_Lib_R	GAGAAAAGAAAACCGCCGATCCTG	Linearizing the pCAW-Sort-Seq plasmid
Ultramer_Gibs_F	GGTGGACAGGATCGGCGGTTTTCTTTTCTCTTCTCAAAAGCTTCCTGCAGTATTC	Amplifying Ultramer® libraries for Gibson Assembly
Ultramer_Gibs_R	GGCTGTTTCGTCCTCACGGACTCATCAGACCGGAAAGCACATCCGGTG	Amplifying Ultramer® libraries for Gibson Assembly

Table S6. Primers with barcodes for demultiplexing sequencing bins:

Name	Sequence
Bin_1_F	**AGTCTCGGCA**ACGGAAGGTAGATTTTACGGC
Bin_2_F	**GATATAGCTC**ACGGAAGGTAGATTTTACGGC
Bin_3_F	**CGTCCGACTT**ACGGAAGGTAGATTTTACGGC
Bin_4_F	**CTCGTGCCTA**ACGGAAGGTAGATTTTACGGC
Bin_5_F	**TAAGATTGCC**ACGGAAGGTAGATTTTACGGC
Bin_6_F	**AGGCCTGACT**ACGGAAGGTAGATTTTACGGC
Bin_7_F	**GTCAATCTTC**ACGGAAGGTAGATTTTACGGC
Bin_8_F	**ATGACGGTAA**ACGGAAGGTAGATTTTACGGC
Bin_9_F	**AGGCTCAAGG**ACGGAAGGTAGATTTTACGGC
Bin_10_F	**GCTCAGTAAT**ACGGAAGGTAGATTTTACGGC
Bin_11_F	**ACGATGAAGT**ACGGAAGGTAGATTTTACGGC
Bin_12_F	**GAGCAGATAT**ACGGAAGGTAGATTTTACGGC
Bin_13_F	**CGATAGCGAG**ACGGAAGGTAGATTTTACGGC
Bin_R_1	TCCTCACGGACTCATCAGAC

Barcodes are represented in bold letters

Supplementary figures:

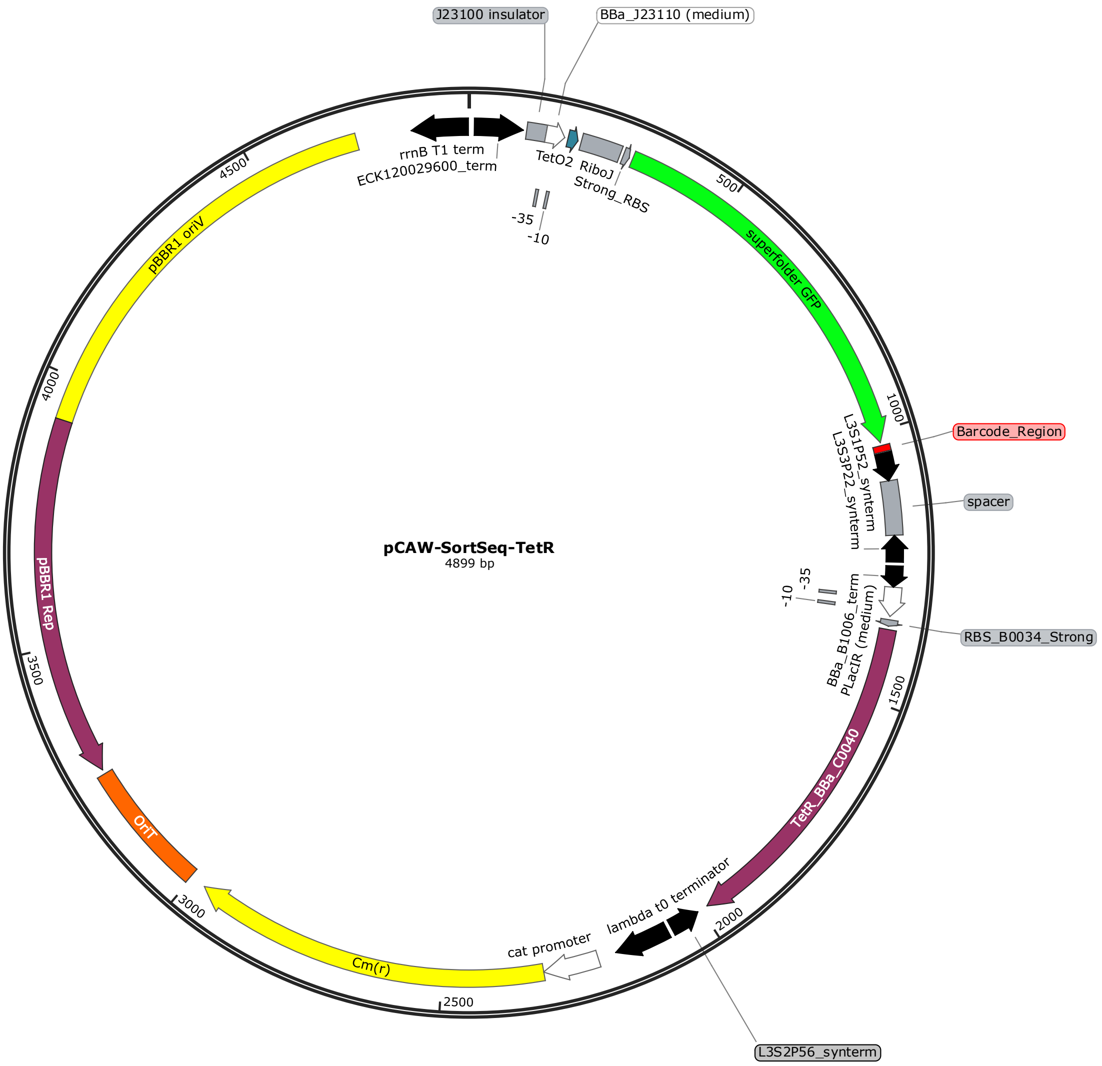

Supplementary Figure S1. The pCAW-SortSeq-TetR plasmid. The plasmid system pCAW-SortSeq encodes a broad-host, low-copy number replication origin (pBBR1 replication origin – 5 to 10 copies) [3], an interchangeable regulatory region where the TFBS is located and placed between a constitutive promoter (BBa_J23110 [4,5]), a superfolder GFP (*sfgfp*) fluorescent reporter gene [6], as well as a *tetr* gene. The *tetr* gene is derived from the original Tn10 transposon [7,8] under the control of a low-strength constitutive promoter (pLac promoter variant developed by [9]).

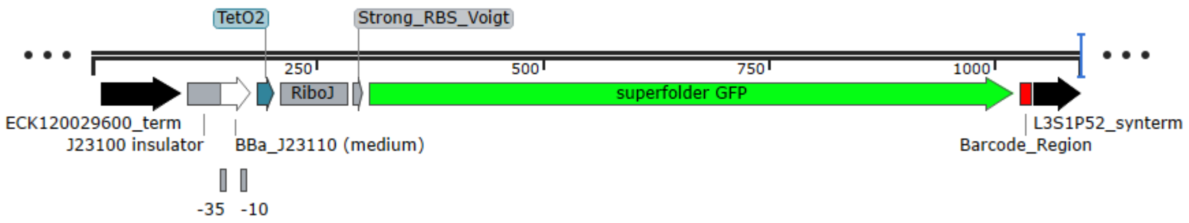

Supplementary Figure S2. The repression measurement module of the pCAW-SortSeq-TetR plasmid. This module encodes the promoter insulator *J23100_Insulator* upstream of the *BBa_J23110* constitutive promoter from [10]. Promoter insulators are transcriptional terminators that alleviate contextual effects of upstream sequences on promoter regions. Bold underlined letters represent -35 and -10 boxes. The constitutive promoter of this module is the *BBa_J23110* promoter from the iGEM registry of parts (http://parts.igem.org/Part:BBa_J23110)[4,5], which has medium promoter strength. We placed the *tetO2* sequence[11] at the +10 position relative to the *sfgfp* transcription start site, which is the optimal distance for synthetic repression [12]. The transcriptional insulator RiboJ has been described in [13]. It is a synthetic ribozyme that removes 5'UTR interferences with variable TFBS sequences in the mRNA by self-cleavage with high efficiency[14]. We obtained the strong synthetic RBS sequence from ref. [15], the reporter gene superfolder GFP (*sfgfp)* from ref. [6], and the strong synthetic transcriptional terminator (*synterm*) from ref. [16]**.**

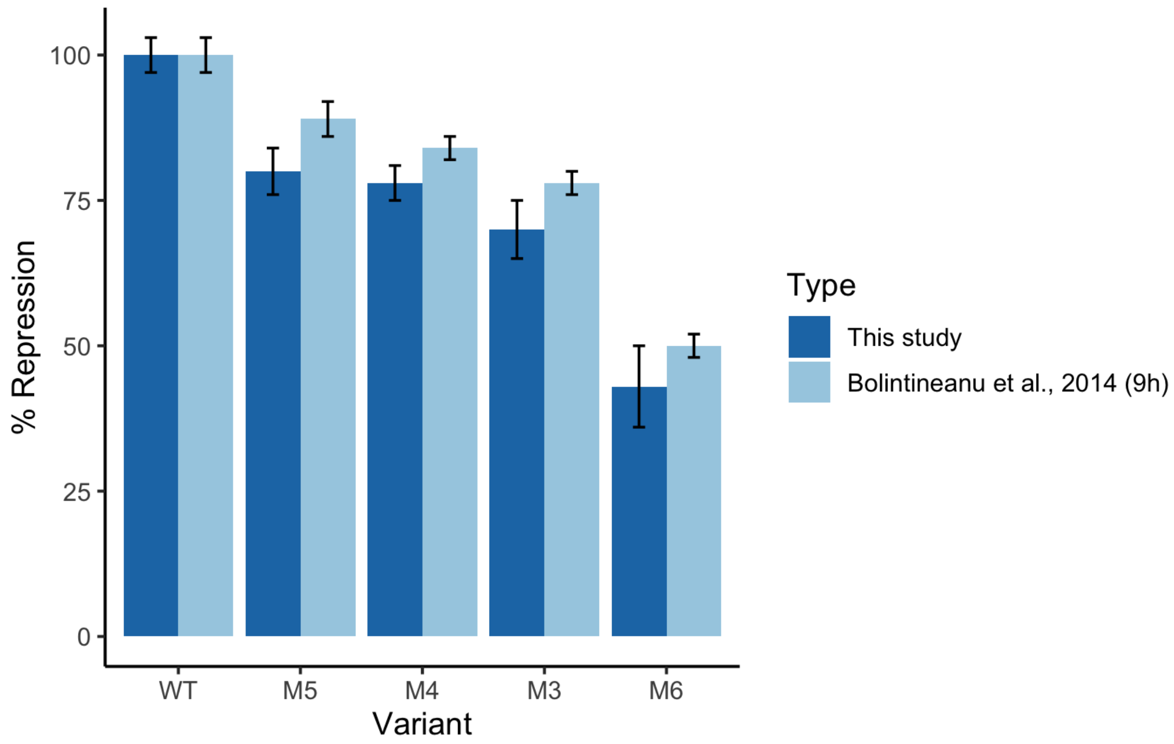

Supplementary Figure S3. Validating the pCAW-SortSeq-TetR plasmid with *tetO2* mutants. We validated the pCAW-SortSeq-TetR plasmid with *tetO2* mutants by measuring the percentage of repression (vertical axis) for each of the five *tetO2* variants (WT, M5, M4, M3, M6, horizontal axis). For calculating percentages of repression, we measured GFP fluorescence distributions for each variant in triplicate in a flow cytometer, divided the mean fluorescence (over triplicate measurements) of each variant by the mean of the WT *tetO2*, and multiplied by 100. Light blue bars represent the data from a previous study[17] characterizing each of the five TetR TFBS variants. Dark blue bars correspond to measurements obtained in the present study. Error bars represent standard deviations among replicates. Source data are provided with this paper.

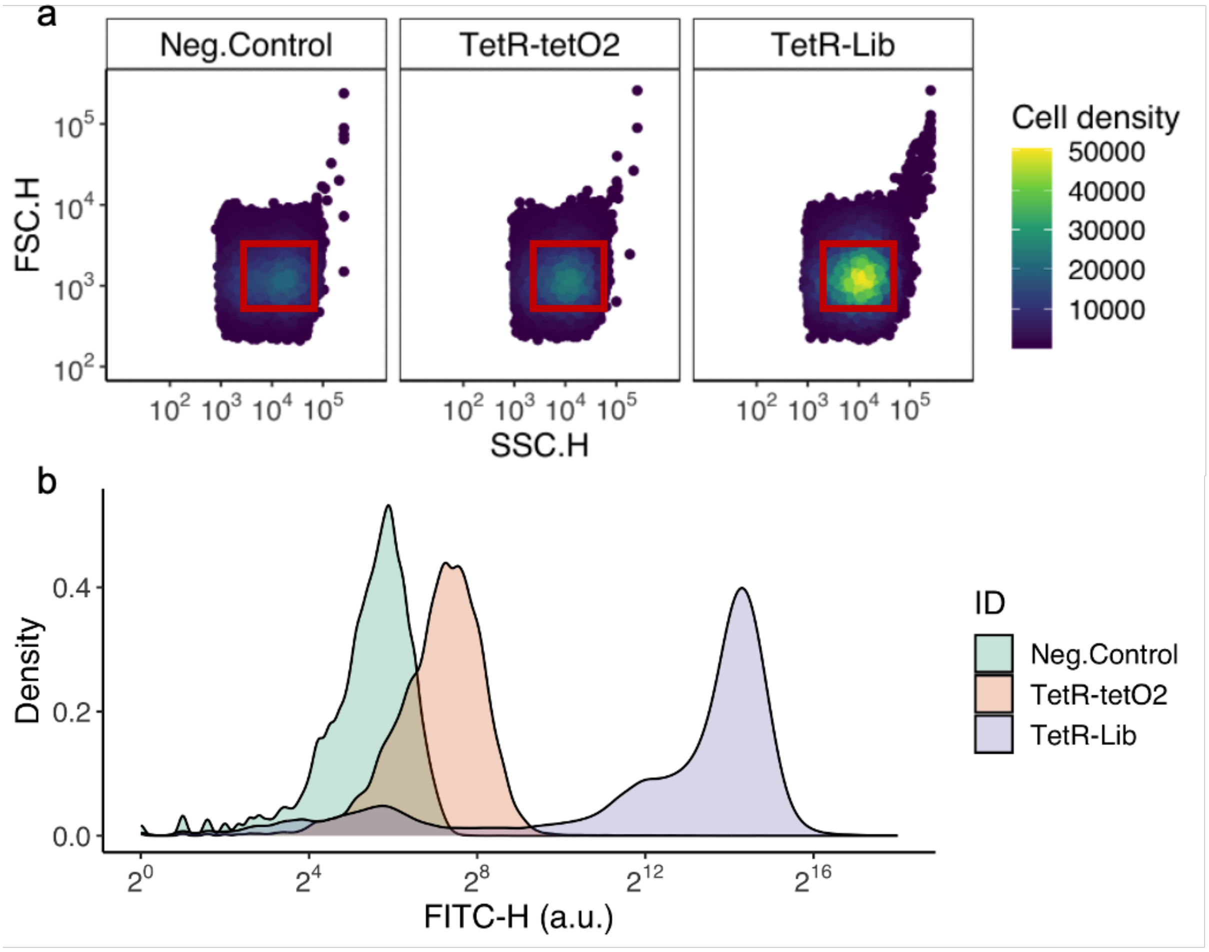

Supplementary Figure S4. Flow cytometry gating and fluorescence expression levels for controls and library. a. Gating of three representative cell populations. We measured the forward (FSC.H, vertical axis) and side scatter (SSC.H, horizontal axis) for 200,000 cells per sample. Inside each grid, we depict individual cells as circles. Heatmap colors represent population densities (see color legend). From left to right: cells harbouring a negative control plasmid (pCAW with promoterless GFP), a positive *tetO2* control (pCAW with the wild-type *tetO2* instead of the mutant library), and *tetO2* variants (pCAW with variant library). All populations were grown in the absence of anhydrotetracycline. The red box represents the region of each scatter plot where cell density was the highest, from which cells were sorted in subsequent experiments. Source data are provided with this paper. **b. Fluorescence distributions of three cell populations transformed with different plasmids.** Density plot with the fluorescence distribution for the same three samples described above (see color

legend). The horizontal axis represents the range of values for GFP fluorescence as FITC-H (arbitrary units, note the log_2 scale). The vertical axis represents the relative frequency of observations for each fluorescence value on the horizontal axis. Density smoothing was performed using a Gaussian kernel function to create a smooth density plot – *ggplot2* package[18]. Source data are provided with this paper.

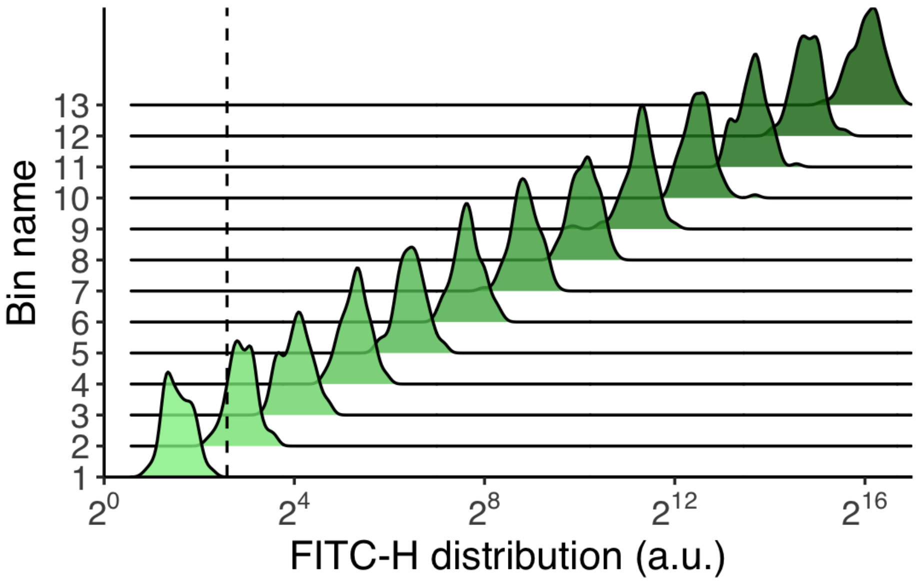

Supplementary Figure S5. Distribution of fluorescence levels after sorting of cells expressing the library into fluorescence bins. The distribution of fluorescence values for each bin is shown as individual density plots. The color gradient represents changes in GFP expression levels (as quantified in the FITC-H channel, arbitrary units) across the horizontal axis, with lighter green corresponding to low GFP expression (and thus higher repression) and darker green corresponding to higher GFP expression (and thus weaker repression). The vertical dashed line represents the autofluorescence threshold based on the obtained geometric mean calculated over the fluorescence distribution for the negative control population. We determined the distribution of values for each bin from a population of 200,000 cells. Source data are provided with this paper.

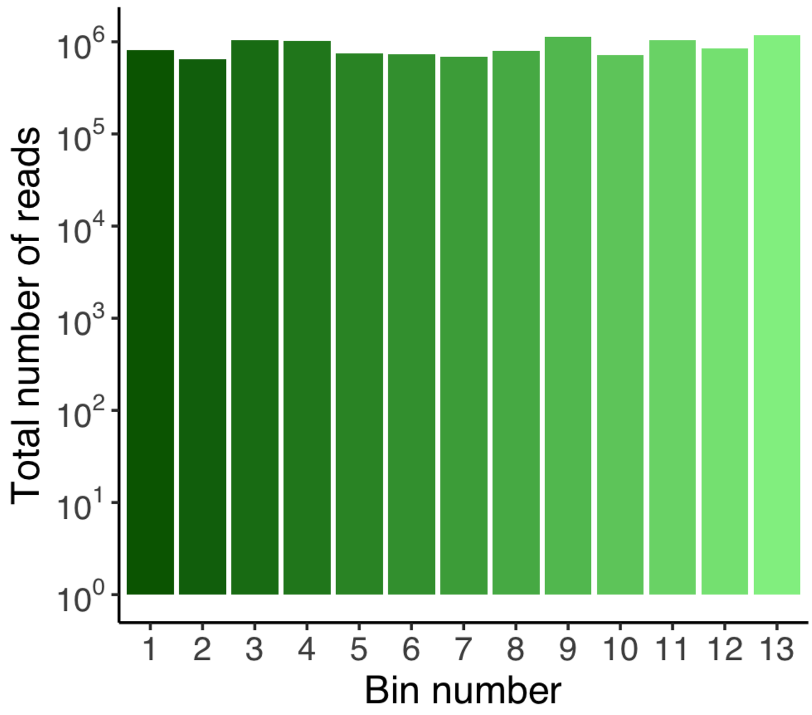

Supplementary Figure S6. Distribution of sequence reads per bins. Gradient bar colours depict GFP expression levels across bins. The total number of sequence reads in each bin is represented on the vertical axis on a logarithmic scale. Source data are provided with this paper.

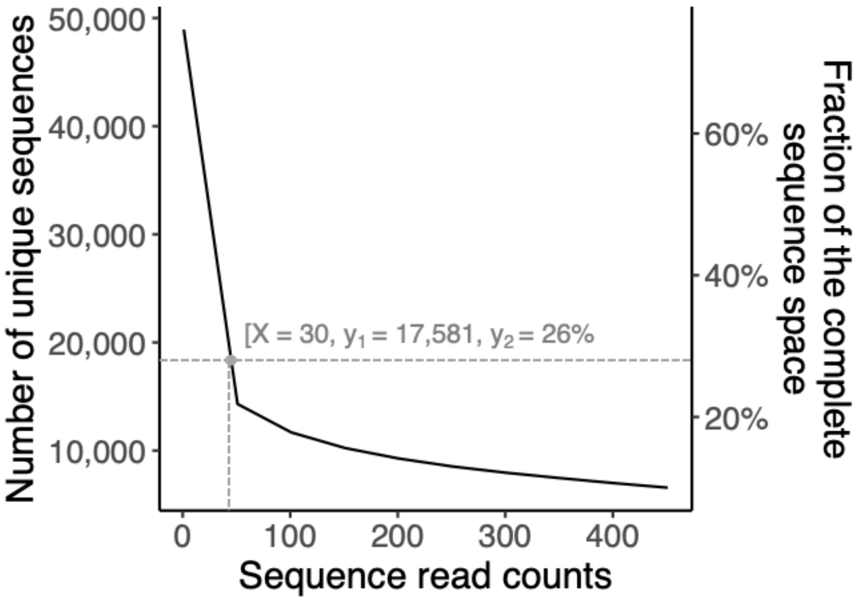

Supplementary Figure S7. Number of genotypes obtained by choosing different sequencing depth (read count) cutoffs. The plot shows the number of unique sequences obtained when applying different sequencing depth (read count) cutoffs. The x-axis represents different read count thresholds in the interval (1, 500), indicating the minimum number of required reads across all bins to include a genotype in our analysis. The left y-axis shows the absolute number of unique sequences, while the right y-axis expresses this number as a percentage of the complete sequence space of 4^8 sequences. When the read count threshold equals one, the number of unique sequences is 48,937 genotypes, or approximately 75% of genotype space. The dashed line represents the threshold of 30 reads we used in our analysis, leading to 17,851 or 26% of genotype space. Source data are provided with this paper.

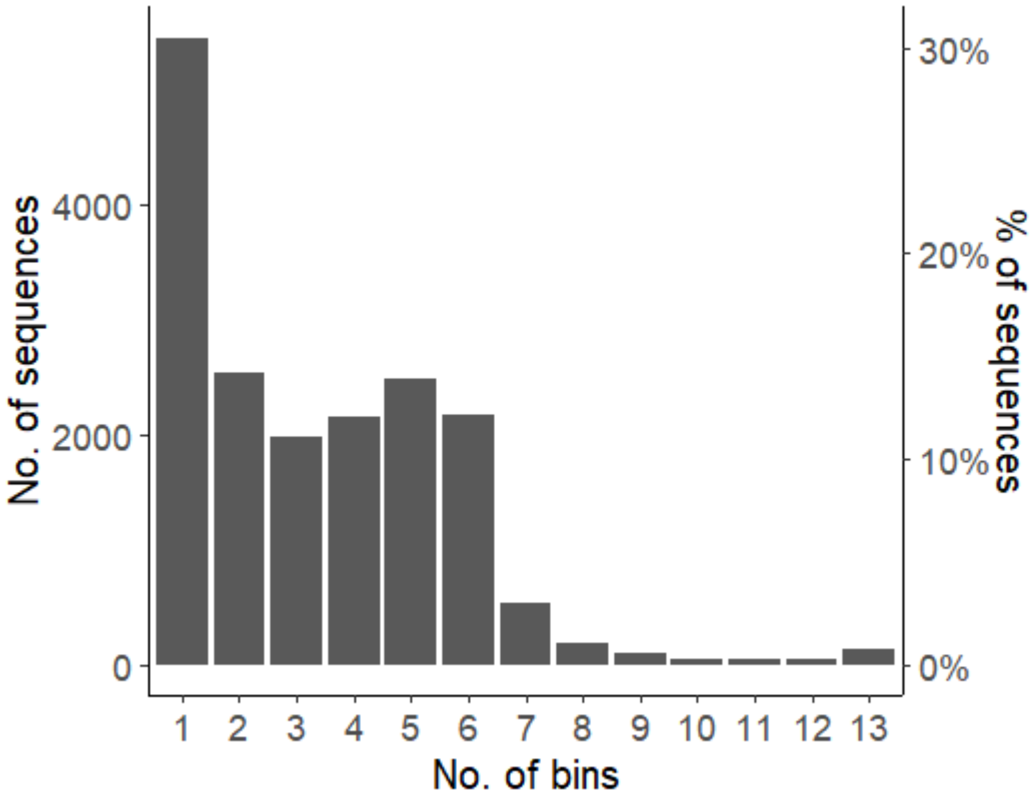

Supplementary Figure S8. Number of bins per genotype. The figure shows a histogram of the distribution of the number of bins (horizontal axis) into which each TFBS variant was sorted. The secondary vertical axis on the right shows the same information, but as a percentage of the total number of sequences (100% =17,851 sequences). 32% of sequences were sorted only into a single bin; 65% of sequences were sorted into 2 to 6 bins. Source data are provided with this paper.

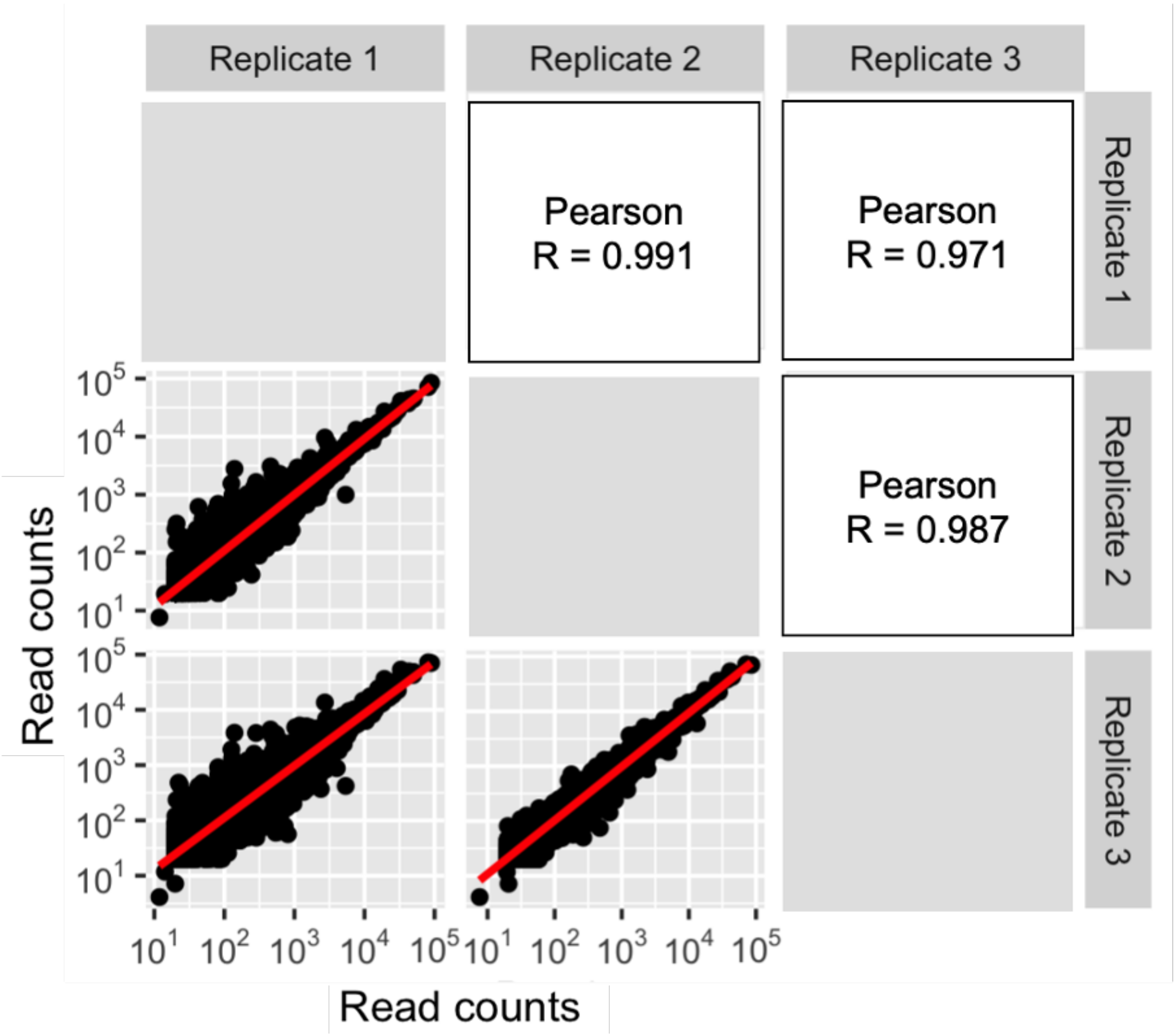

Supplementary Figure S9. Correlation of read coverage among replicates of the *tetO2* mutant library. Note the logarithmic scale in all panels. Correlation plots are represented as scatter plots in the lower panels, the red line in each plot is the x=y line. The R in the upper figure panels represent the Pearson correlation coefficients calculated for each pair of replicates. Source data are provided with this paper.

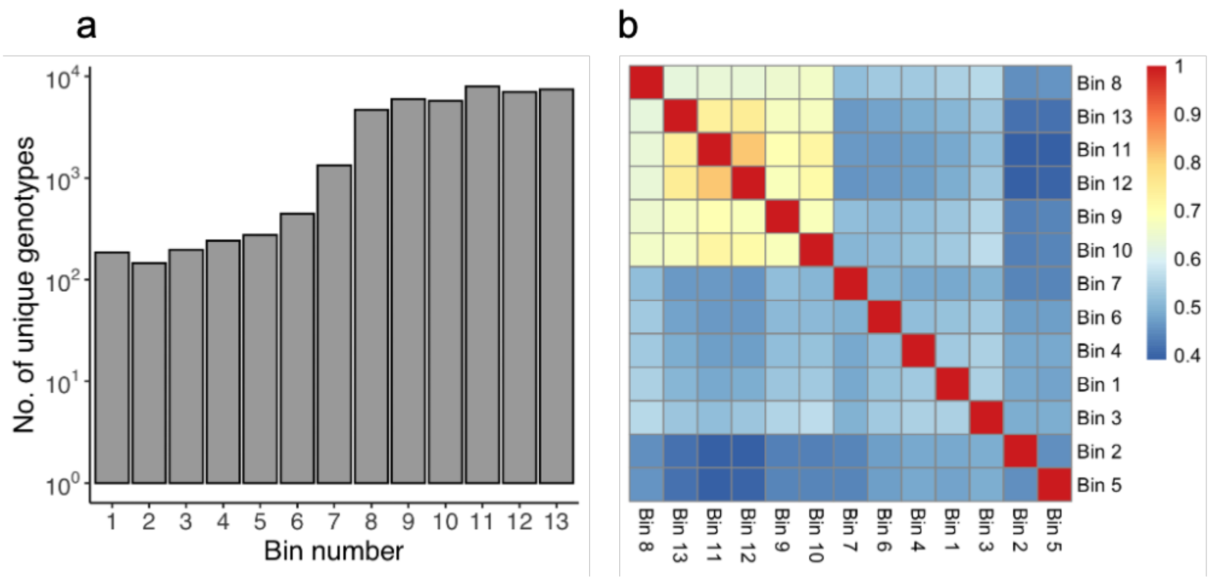

Supplementary Figure S10. Genotypes per fluorescence bin and overlaps between them. a. Number of individual genotypes per bin. The vertical axis shows the number of unique genotypes per bin for each of the 13 fluorescence bins (horizontal axis) into which we sorted cells. Low fluorescence bins correspond to TFBS variants conferring strong repression, of which there are fewer, hence, there are also fewer unique genotypes in these bins. Source data are provided with this paper. **b. Heatmap of the fraction of genotypes shared between different bins.** The data is represented as a symmetric matrix of pairwise fractional overlaps calculated using the Jaccard index coefficient [19,20] between all possible pairs of the13 bins. Each row and each column corresponds to a bin. Red values represent complete overlap between bin sequences and dark blue values represent the minimum overlap observed (40%). We ordered and clustered bins using an Euclidean distance with a complete-linkage clustering method. The Euclidean distance measures the similarity or dissimilarity between bins, and the complete-linkage clustering merge clusters based on the distance between their farthest points. Note that overlaps do not consider read counts. For example, two bins might share 40% of their sequences but the read count between the same sequence in each bin might differ by orders of magnitude. Note also that the overlap is greatest for high fluorescence bins, which also contain the most genotypes (panel a). The higher genotype overlap between higher bins (Bins 8-13)

can be explained by a higher number of cells sorted into these bins in comparison to lower bins.

Source data are provided with this paper.

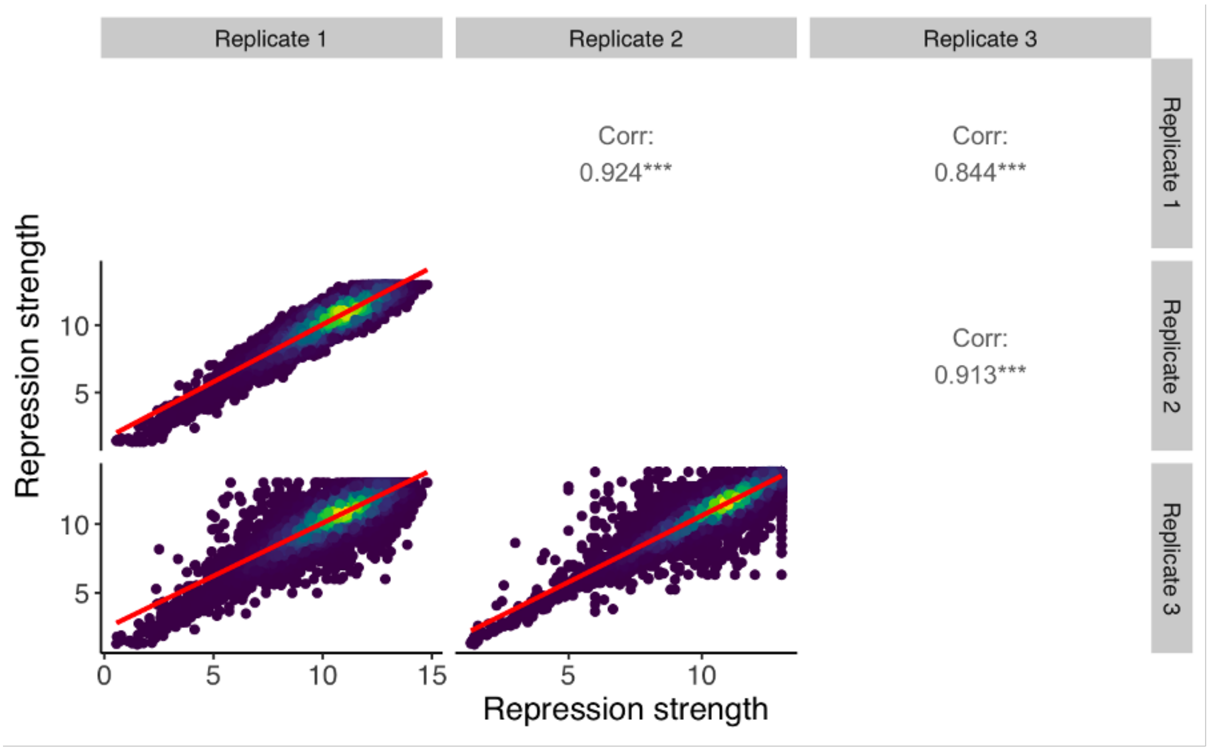

Supplementary Figure S11. Reproducibility of estimated repression levels. Figure Legend. Fluorescence-based sorting methods are inherently noisy, especially at the highest and lowest bounds of a fluorescence distribution. To assess how such variability could impact our estimate of repression strengths, we computed the correlation of repression strength derived from the fluorescence data between the three replicates we performed. Each point in each scatter plot represents a distinct genotype, plotted according to its repression strength in the two replicates indicated in the grey rectangles on the top and to the right of the plot matrix. The color gradient in the plots represents the density of genotypes (purple: low density; yellow: high density). The red line in each plot represents a linear model's fit to the data. Pearson correlation coefficients for the replicates are high (R=0.84 to 0.92, upper triangle of the plot matrix) and demonstrate high reproducibility of our estimates, although somewhat lower than for read counts (**Supplementary Figure S9**). Source data are provided with this paper.

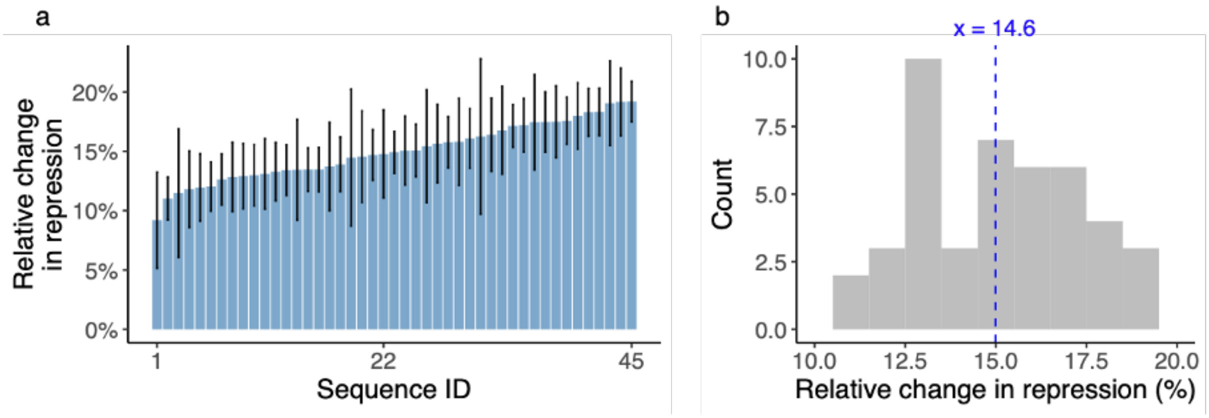

Fig. S12. Validation of regulation strength differences in the lowest fluorescence bins (strongest repression). a. Increase in repression strength expressed as a percentage relative to the wild-type, for 45 sequences with the highest repression strength, measured using a plate reader. The selected sequences repressed gene expression substantially more strongly than the WT, with a mean relative repression strength increase of 14.6% ± 3.2%. **b. Histogram of the relative change in repression for the 45 selected sequences.** The vertical dashed blue line indicates the mean repression strength increase relative to the wild-type of 14.6%. This difference is significantly greater than zero (Welch one-sample t-test, t = -55.737, df = 44, p-value < 2.2 x 10^{-16}). Source data are provided with this paper.

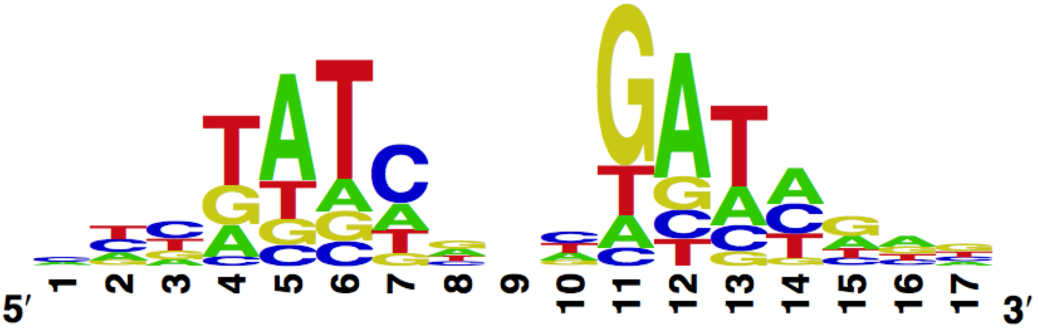

Supplementary Figure S13. DNA Sequence logo obtained by a previous study. Using the MITOMI[21] in vitro technique, the 2011 iGEM team of the École polytechnique fédérale de Lausanne (EPFL) studied the DNA binding landscape of the wild-type TetR sequence. To do so, they designed and generated a library of double-stranded DNA sequences that covered all possible single base substitutions within the *tetO2* binding site sequence. Based on that library, the team measured the dissociation constants of each variant relative to the average constant of all the *tetO2*-like variants of the library. Then, they determined the specificity of TetR for the binding site variant sequences, expressed as a position-weight matrix (PWM). The figure shows the corresponding DNA sequence logo. Original figure available at https://2011.igem.org/Team:EPF-Lausanne/Our_Project/TetR_mutants/MITOMI_data).

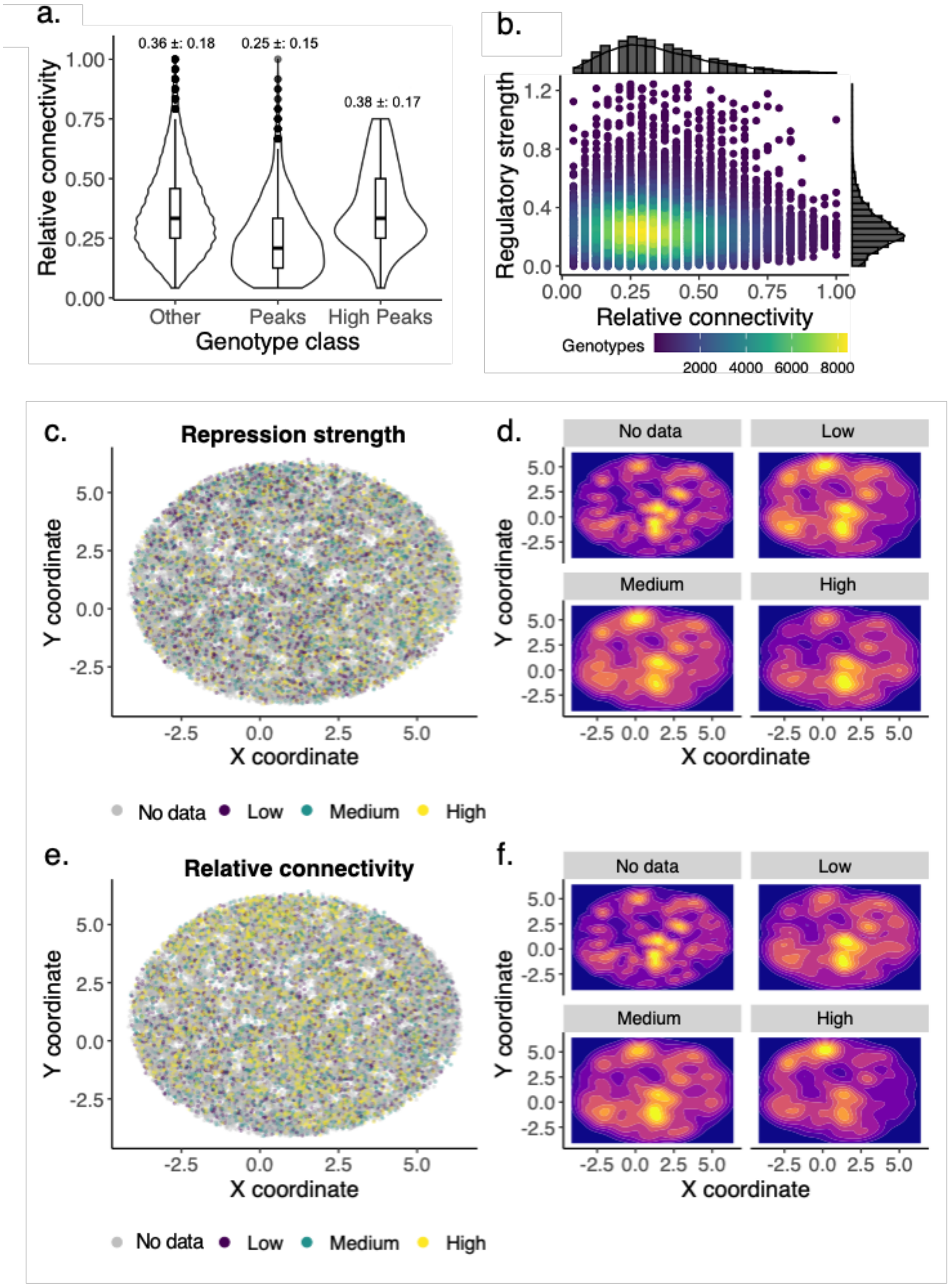

Supplementary Figure S14. Quantitative analysis of TetR landscape sparsity. a. Distribution of relative connectivity among genotypes. Violin plots augmented with embedded boxplots illustrate the distribution of relative connectivity, i.e., the ratio of the empirically observed number of adjacent genotypes to the theoretical maximum number of 24(=8×3) within a fully connected network. Genotypes are stratified into the three categories: "other" (non-peak genotypes), "peaks" (excluding high peaks), and "high peaks". Mean relative

connectivities and standard deviations are shown above each categorical division, revealing lower relative connectivity in peaks relative to non-peak genotypes (Two-sample Welch's t-test, p-value = 4.6 x 10^{-177}, N1= 15,671; N2 = 2,092). High peaks exhibit superior connectivity relative to other peak genotypes (Two-sample Welch's t-test, p-value = 3.1 x 10^{-144}, N1 = 2,092; N2 = 58). High peaks exhibit comparable or superior connectivity relative to non-peak genotypes (Two-sample Welch's t-test, p-value = 1, N1 = 15,671; N2 = 58). Source data are provided with this paper. **b. Weak association between connectivity and repression strength.** Scatterplot of relative connectivity against repression strength. The density of genotypes (circles) in the scatterplot area is represented by a color gradient from purple (low density) to yellow (high density). The weak association (Pearson correlation, R = -0.13; t = -23.888; degrees of freedom (df) = 31,973; p-value < 2.2e-16) indicates that strongly regulating genotypes are not necessarily densely connected. Marginal histograms adjacent to the scatterplot quantify the distributions of repression strength and relative connectivity. Source data are provided with this paper. **c. Visualization of TetR landscape based on repression strength.** Visual overview of the TetR landscape with genotypes color-coded according to repression strength (low strength: purple; medium: green; high: yellow; grey: genotypes with missing data). To enhance clarity, edges between genotypes are not shown. The landscape is projected onto a 2D space through a force-directed algorithm[22], with axes representing arbitrary units. Source data are provided with this paper. **d. Spatial distribution of repression strengths.** Each panel shows a density contour plot of the distribution of repression strength scores of genotypes within one of four repression strength categories (No data [genotypes with missing data], low, medium, high). It indicates the density of genotypes within each of the four categories through a color gradient from blue (low density) to yellow (high density). Source data are provided with this paper. **e. Visualization of TetR landscape based on relative connectivity.** Analogous to panel c, but for relative connectivity. Color-codes indicate relative

connectivity (see color legend). Source data are provided with this paper. **f. Spatial distribution of relative connectivity.** Like panel d, but for four categories of relative connectivity (no data, low, medium, high). Source data are provided with this paper.

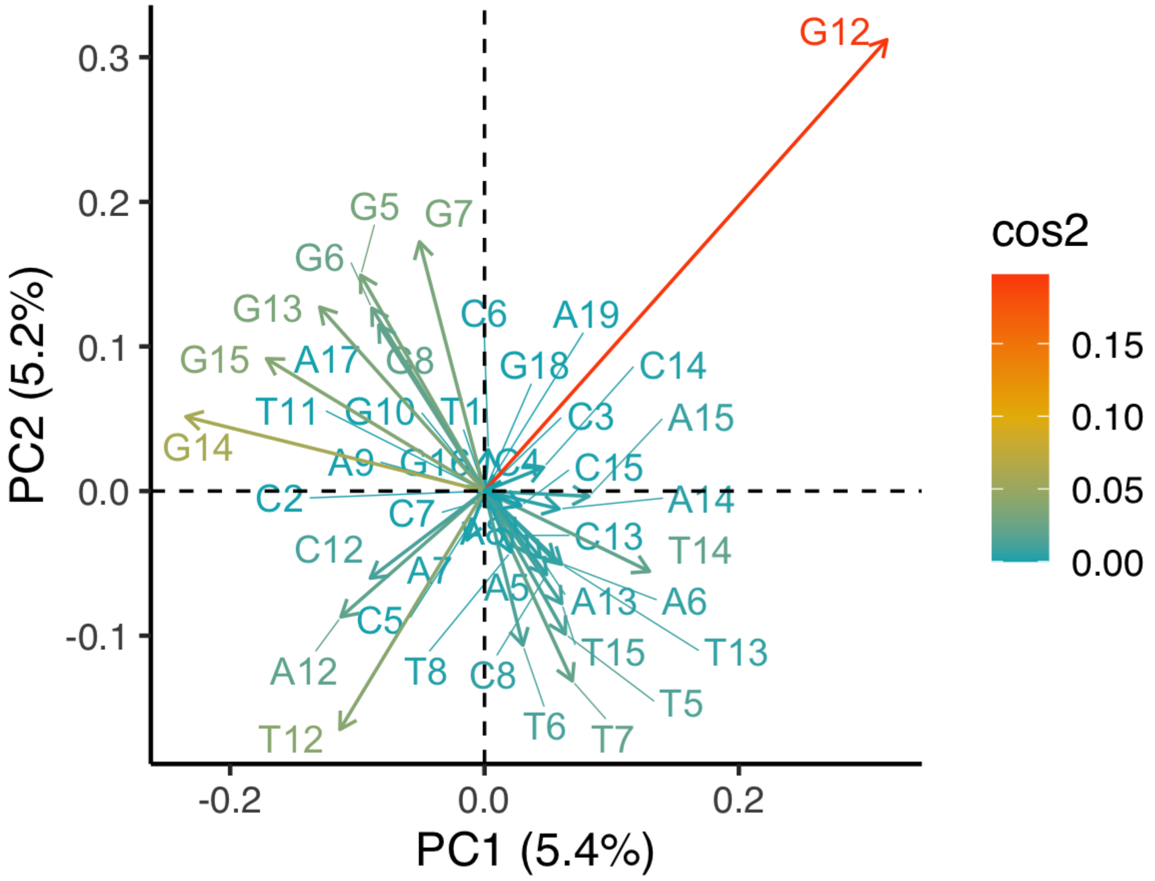

Supplementary Figure S15. The contribution of individual *tetO2* nucleotides in principal component analysis. A PCA (Principal Component Analysis) contribution plot is a way to visualize the relative importance of different variables to the variation observed in the data. In this plot, the contribution of each variable is expressed as the square of the cosine of the angle (cos2) between the variable's vector (column representing the presence/absence of a base letter at each position in the sequence) and each principal component axis. This quantity is represented as an arrow that indicates the correlation of the variable with PC1 and PC2, the two principal components that capture the largest amount of variation in the data. Both length and color of the arrow represent the contribution of the variable to the variation observed in the data. A high cos2 value (red) indicates that the variable is strongly correlated with the principal

component, and therefore makes a large contribution to the variation observed in the data. A low cos2 value (blue) indicates that the variable is weakly correlated with the principal component, and therefore makes a small contribution to the variation observed in the data. The arrow size represents the importance of the variable's contribution relative to other variables in the plot. Each nucleotide is represented as a base letter (A, T, C, G) followed by a number that indicates the position of that base in the binding site sequence (e.g., G12 stands for a guanine at position 12 of the binding site). Source data are provided with this paper.

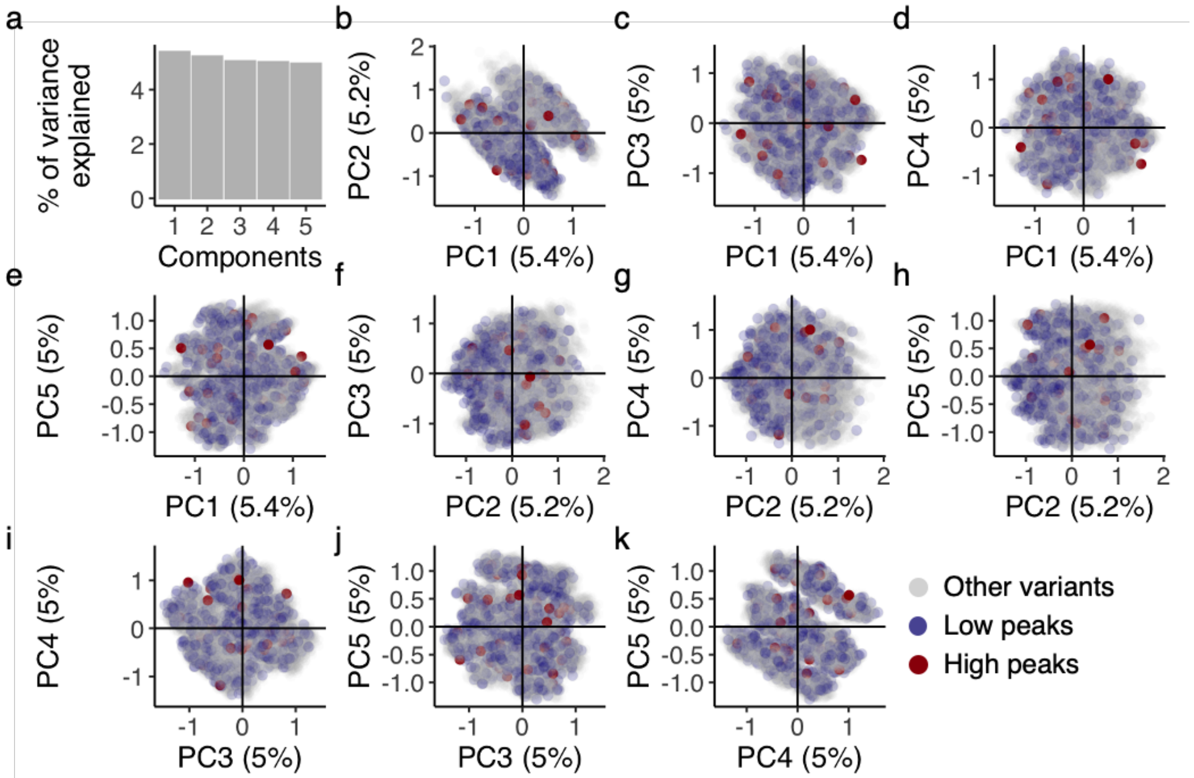

Fig. S16. Principal component analysis (PCA) of peak and non-peak genotypes. a. The proportion of variation explained by each principal component. Given the combinatorial complexity and high dimensionality of our genotype space, each principal component accounts for only a small fraction of the genetic variation. Source data are provided with this paper. **b-k. PCA plots for all pairs of the first five principal components.** We performed PCA after one-hot encoding all genotypes in the landscape. Each circle represents one of the 17,851 variants in the landscape, with colors indicating the class assigned to each genotype: non-peak variants are shown in grey, low peaks in blue, and high peaks in red. Source data are provided with this paper.

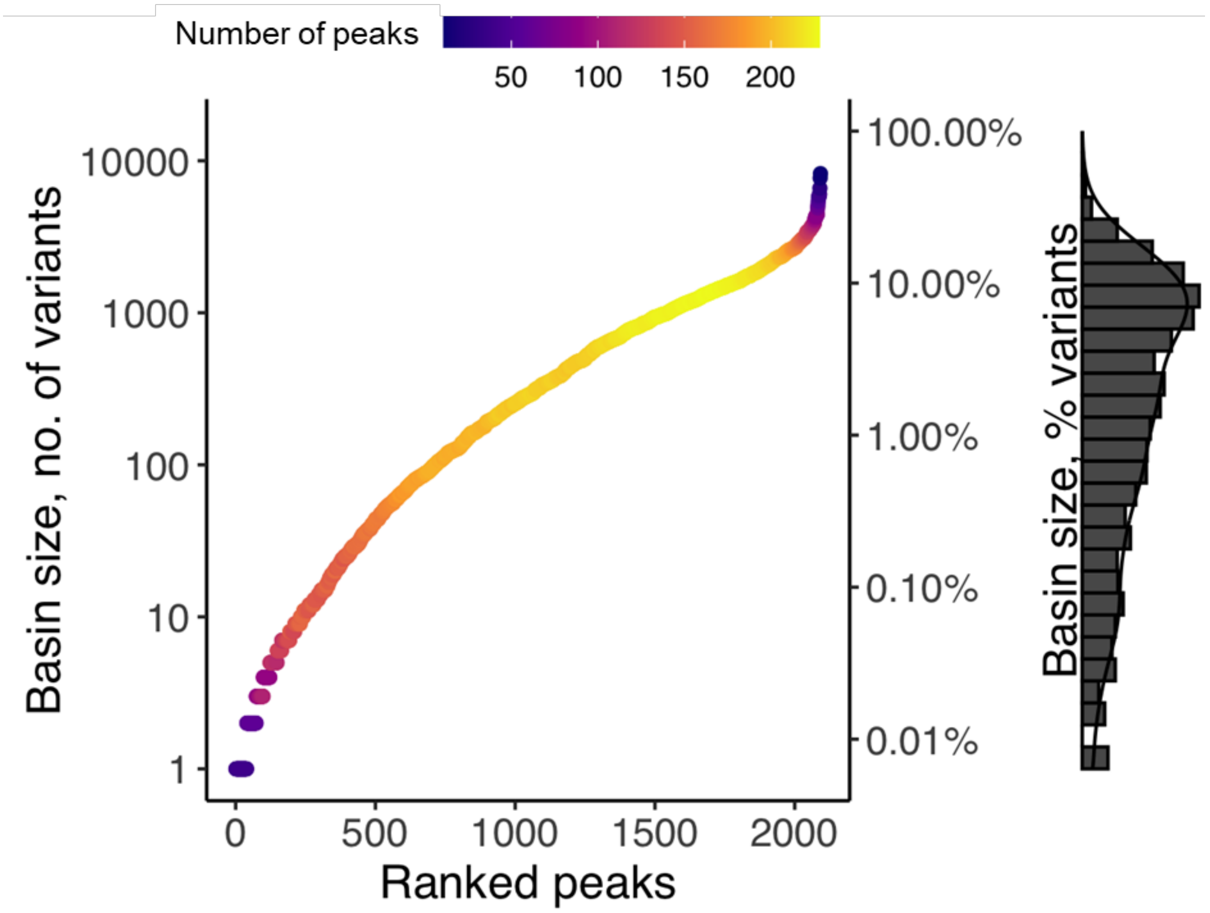

Supplementary Figure S17. The distribution of basin sizes among peaks. Peaks are ranked along the horizontal axis according to the size of their basins of attraction (vertical axis). The secondary vertical axis on the right represents basin size as a percentage of variants (100% =17,765 variants). Heatmap colors represent the number of peaks at each position of the ranked scatterplot (see color legend). The marginal histogram on the right shows the distribution of basin sizes. Source data are provided with this paper.

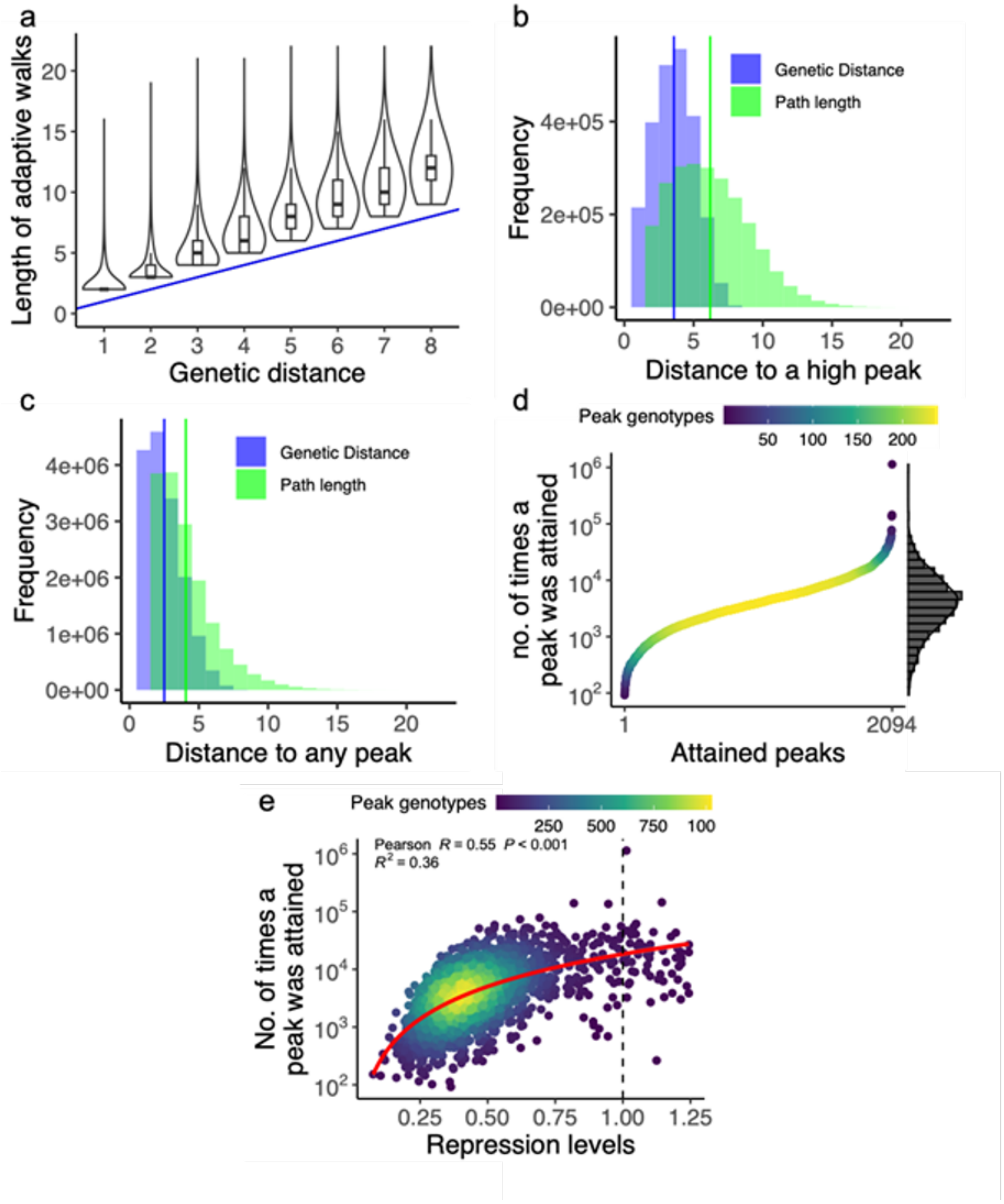

Supplementary Figure S18. Adaptive walks in which each mutational step is chosen with uniform probability among all repression-increasing steps. Data displayed here are based on 1,000 adaptive walks starting from each non-peak genotypic variant (N= 15.671). **a. Adaptive walks leading to high repression peaks are predominantly short.** The vertical axis presents the number of mutational steps in adaptive walks that initiate from a random

variant and converge at a high repression peak. The horizontal axis reflects the shortest genetic distances between the starting variant and the attained peak. The blue line (y=x) signifies the most direct distance to a high repression peak, equated by the genetic distance. Violin plots summarize the shape of distributions with a probability density function. A wider probability density function indicates that a value on the y-axis occurs more frequently, and a narrower density function indicates that a value occurs less frequently. Each box covers the range between the first and third quartiles (IQR). The horizontal line within the box represents the median value, and whiskers span 1.5 times the IQR. Values beyond the 1.5 IQR interval are shown. Adaptive walks were only marginally longer than shortest paths. Source data are provided with this paper. **b. Accessible paths to high repression peaks tend to be short.** The blue histogram shows the distribution of the genetic distances for all pairs of variants and their respective attainable high peaks. The green histogram shows the distribution of the number of mutational steps for the shortest accessible paths between variants and their respective attainable peaks. Source data are provided with this paper. **c. Most accessible paths to any (high or low) repression peak are short.** The blue histogram shows the distribution of the genetic distances for all pairs of variants and their respective attainable peaks. The green histogram shows the distribution of the number of mutational steps for the shortest accessible paths between variants and their respective attainable peaks (high or low). Source data are provided with this paper. **d. Some peaks are attained more frequently than others.** We ranked all peaks along the horizontal axis according to the number of times they are reached across all adaptive walk simulations (N= 15.671×10^3, vertical axis, note the logarithmic scale). The marginal density histogram on the right shows the distribution of the number of times each peak was reached from an individual variant. Heatmap colors represent the number of peaks at each position of the ranked scatterplot (see color legend). Source data are provided with this paper. **e. High peaks tend to be attained more often.** The scatter plot shows the repression

level conveyed by a peak variant (horizontal axis) and the number of times a peak with this repression level was reached across all adaptive walks (N= 15.671 × 10^3, vertical axis, note the logarithmic scale). The dashed vertical line represents the repression level for the wild-type sequence. The red curve represents a semi-logarithmic linear regression line for the data, and the grey shade around it represents its 95% percent confidence interval. R is the linear Pearson correlation coefficient, and R^2 is the goodness of fit of the logarithmic linear regression model (N= 15.671). Heatmap colors represent the number of peaks at each position of the ranked scatterplot (see color legend). Source data are provided with this paper.

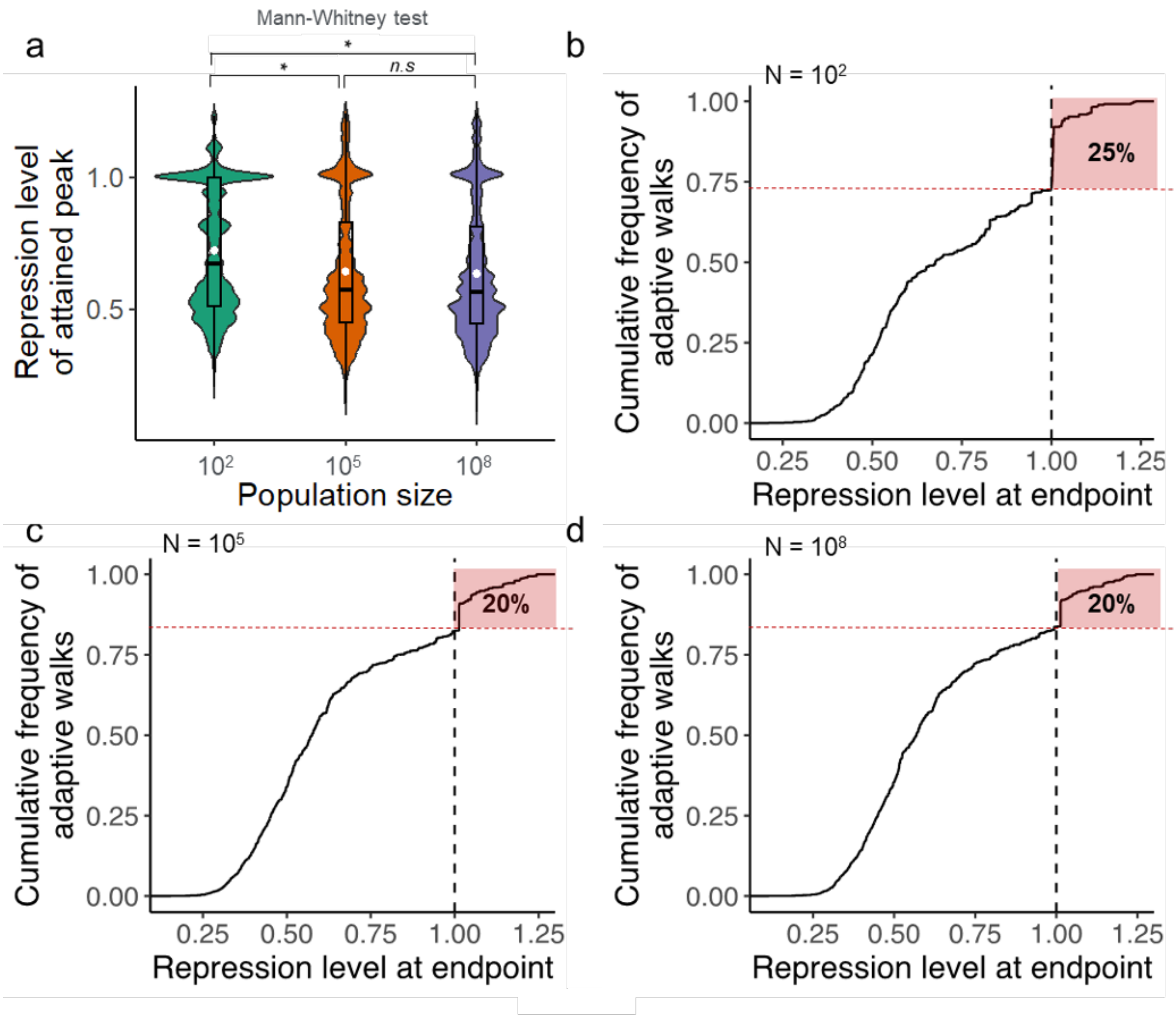

Supplementary Figure S19. Adaptive walks using the Kimura model with different population sizes. Data displayed here are based on 10^6 adaptive walks starting from 10^3 random variants (10^3 random walks per variant) for small ($N=10^2$), medium ($N=10^5$) and large ($N=10^8$) population sizes. **a. Distribution of repression levels of attained peaks for each population size.** Violin plots summarize the shape of distributions for each population size (horizontal axis). Each box covers the range between the first and third quartiles (IQR). The horizontal line within the box represents the median value, and whiskers span 1.5 times the IQR. The white circle inside each boxplot represents the mean of each distribution, which is 0.72±0.34, 0.64±0.25 and 0.63±0.25 (mean ±s.d.) for small, medium, and large population sizes, respectively. The median repression level of attained peaks for small populations (10^2) is significantly higher than that for medium and large populations (two-sided Mann–Whitney

U = 425,000,000, n1 = 10^6, n2 = 10^6 , p = 2.13×10^{-15}). Source data are provided with this paper. **b-d. Small population sizes attain higher peaks.** Each panel shows the cumulative distribution of repression values reached by 10^6 adaptive walks starting from 1,000 random variants (1,000 walks per variant) in the landscape (**Methods**). The population size of each panel is represented by the *N* letter on the upper left of each plot. The dashed vertical line x=1 shows the repression value of the wild type. The area highlighted in red corresponds to the percentages of adaptive walks that reached peaks with repression 1 or greater; 25%, 20% and 20% of adaptive walks for small, medium, and large population sizes, respectively. Source data are provided with this paper.

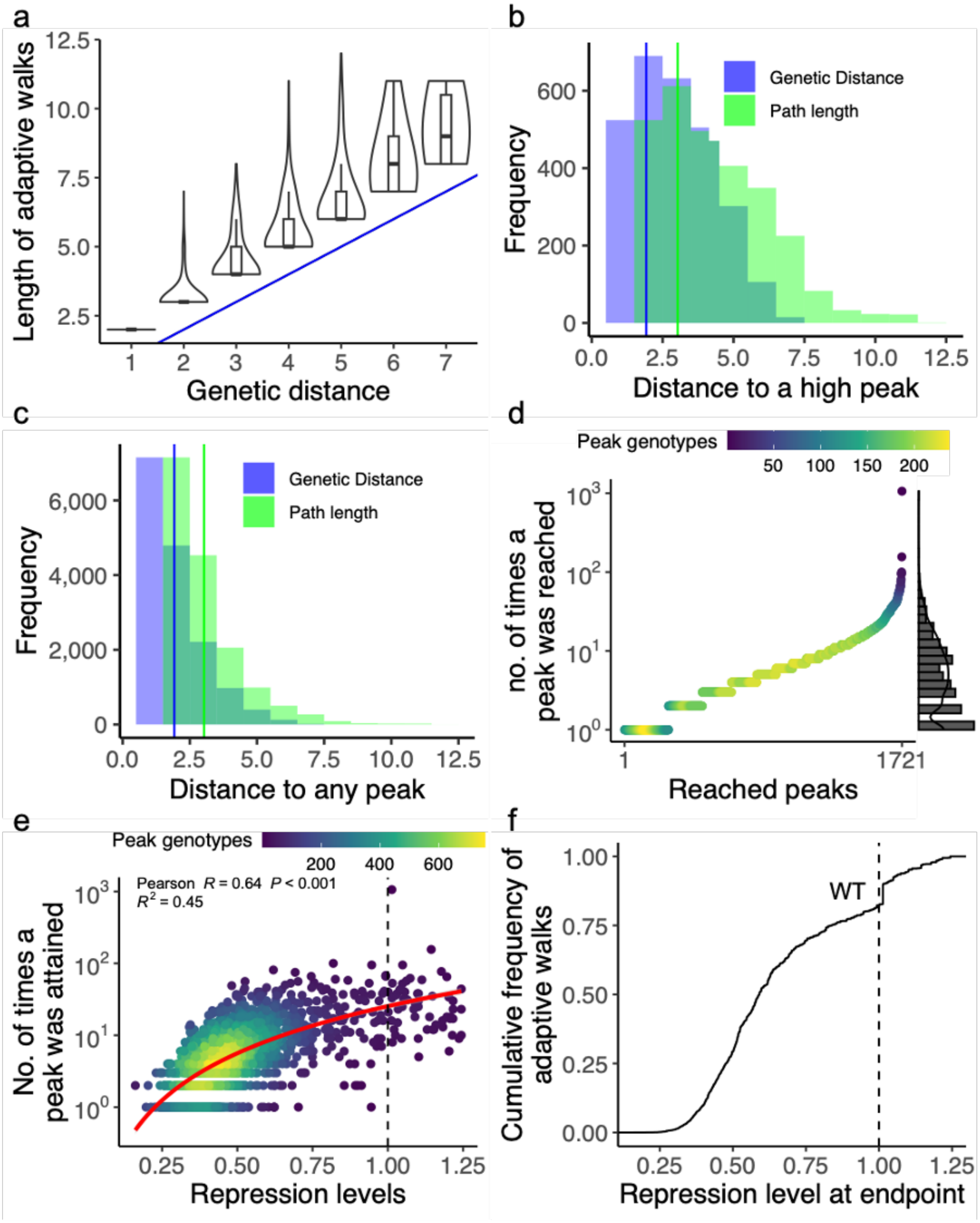

Supplementary Figure S20. Greedy adaptive walk simulations. We performed one greedy adaptive walk starting from each of N= 15,671 non-peak genotypes (**Methods**). Each such walk is deterministic**. a. Adaptive walks leading to high repression peaks are predominantly short.** The vertical axis presents the number of mutational steps in adaptive

walks that initiate from a random variant and converge at a high repression peak. The horizontal axis reflects the shortest genetic distances between the starting variant and the attained peak. The blue line (y=x) signifies the most direct distance to a high repression peak, equated by the genetic distance. Violin plots summarize the shape of distributions with a probability density function. A wider probability density function indicates that a value on the y-axis occurs more frequently, and a narrower density function indicates that a value occurs less frequently. Each box covers the range between the first and third quartiles (IQR). The horizontal line within the box represents the median value, and whiskers span 1.5 times the IQR. Values beyond the 1.5 IQR interval are shown. Adaptive walks were only marginally longer than shortest paths. Source data are provided with this paper. **b. Accessible paths to high repression peaks tend to be short.** The blue histogram shows the distribution of the genetic distances for all pairs of variants and their respective attainable peaks. The green histogram shows the distribution of the number of mutational steps for the shortest accessible paths between variants and their respective attainable peaks. Source data are provided with this paper. **c. Most accessible paths to any (high or low) repression peak are short.** The blue histogram shows the distribution of the genetic distances for all pairs of variants and their respective attainable peaks. The green histogram shows the distribution of the number of mutational steps for the shortest accessible paths between variants and their respective attainable peaks (high or low). Source data are provided with this paper. **d. Some peaks are attained more frequently than others.** We ranked all peaks along the horizontal axis according to the number of times they are reached across all adaptive walk simulations (N= 15.671, vertical axis, note the logarithmic scale). The marginal density histogram on the right shows the distribution of the number of times each peak was reached from an individual variant. Heatmap colors represent the number of peaks at each position of the ranked scatterplot (see color legend). Source data are provided with this paper. **e. High peaks tend to be reached more often.** The scatter plot shows the repression

level conveyed by a peak variant (horizontal axis) and the number of times a peak with this repression level was reached across all adaptive walks (N= 15.671, vertical axis, note the logarithmic scale). The dashed vertical line represents the repression level for the wild-type sequence. The red curve represents a semi-logarithmic linear regression line for the data, and the grey shade around it represents its 95% percent confidence interval. R is the linear Pearson correlation coefficient, and R^2 is the goodness of fit of the logarithmic linear regression model (N= 15.671). Heatmap colors represent the number of peaks at each position of the ranked scatterplot (see color legend). Source data are provided with this paper. **f. High repression peaks are attainable through adaptive evolution.** The panel shows the cumulative distribution of repression values reached by 10^3 adaptive walks starting from each non-peak variant in the landscape (Methods). The dashed vertical line at x=1 shows the repression value of the wild type. Only 20% of adaptive walks reached a repression value of 1 or higher. Source data are provided with this paper.

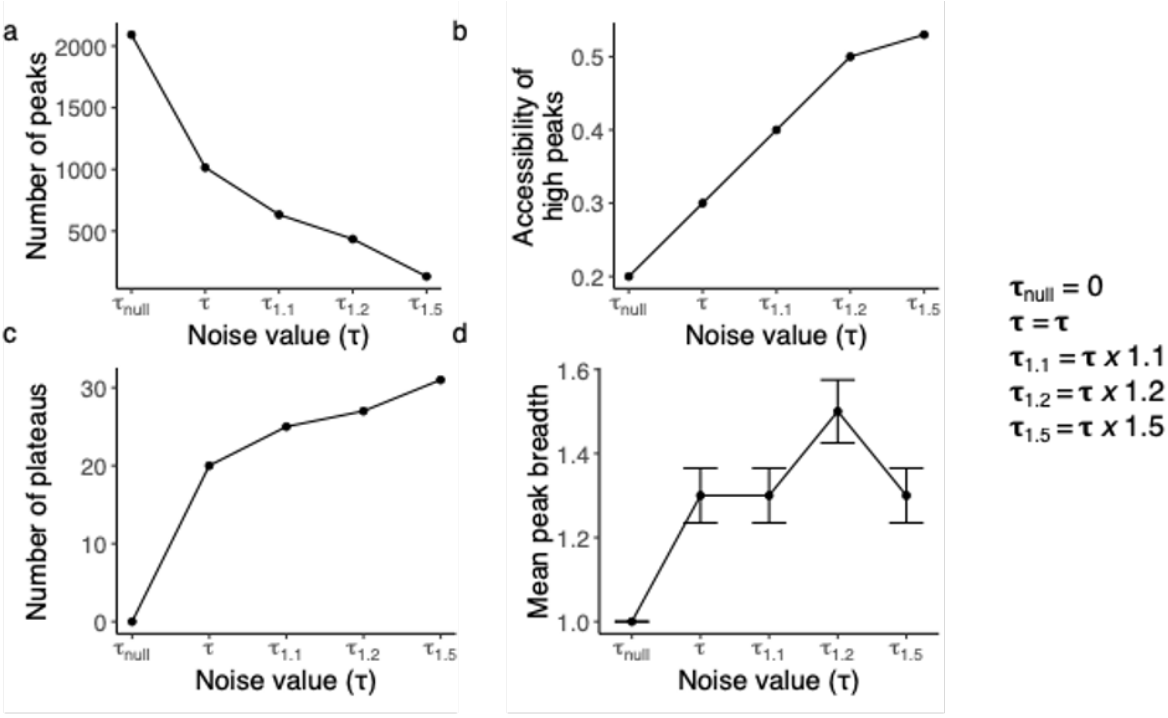

Supplementary Figure S21. The effect of experimental measurement noise on landscape features. In each panel, the horizontal axis shows varying simulated levels of experimental noise in measuring repression strengths that we used to determine how various landscape features (vertical axes) depend on such noise. Specifically, τ_{null} reflects the assumption that all measurements are noise-free. Other noise values (τ to $\tau_{1.5}$), legend on right-hand side) depend on τ, the standard deviation of a genotype's measured repression strengths across the three replicate experiments we performed (**Methods**). In the plots, the symbol τ corresponds to the actual experimental noise estimated from the data. Increasing experimental noise **a)** reduces the number of peaks, **b)** increases the accessibility of high peaks, **c)** increases the number of plateaus (cluster of connected neighboring peaks, **Methods**) and **d)** increases mean peak breadth. Peak breadth reflects the number of peaks composing a plateau and the mean peak breadth, the average number of peaks in each plateau. Error bars depict the diversity in the number of peaks composing individual plateaus. Source data are provided with this paper.

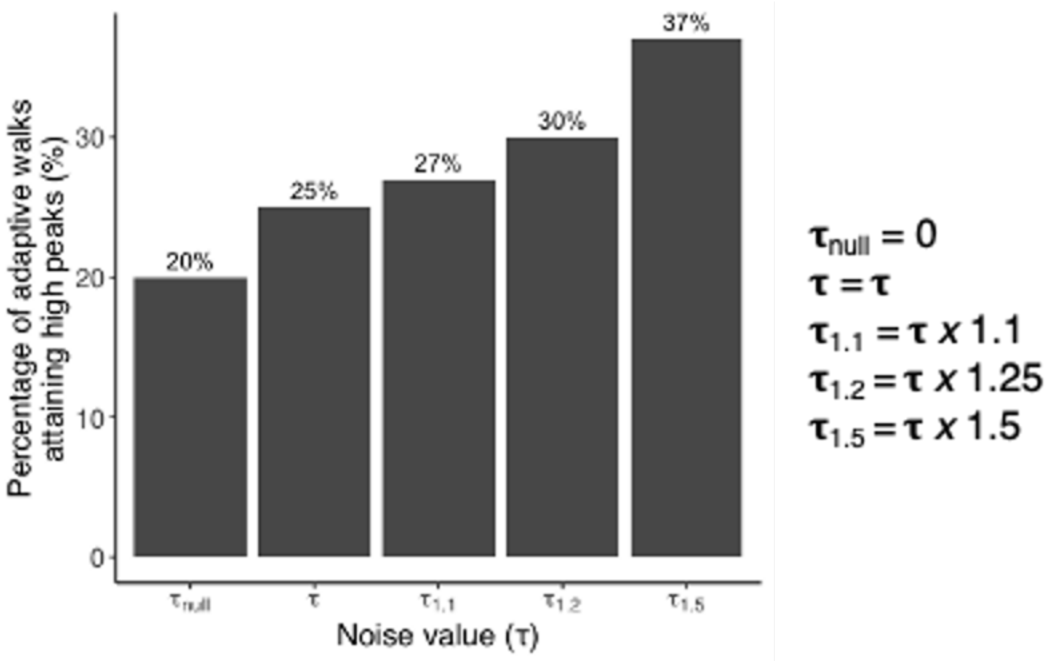

Supplementary Figure S22. The effect of experimental measurement noise on the accessibility of high peaks. The bar plot shows the percentage of adaptive walks (vertical axis, out of 10^6 walks, 10^3 adaptive walks from the same 10^3 starting genotypes at each noise value) reaching high peaks on landscapes generated with different noise levels (horizontal axis, Supplementary Materials 11). Specifically, τ_{null} corresponds to the assumption that all measurements are noise-free. Other noise values (τ to $\tau_{1.5}$), legend on right-hand side) depend on τ, the standard deviation of a genotype's measured repression strengths across the three replicate experiments we performed (**Methods**). As noise increases, the landscape becomes smoother (**Supplementary Figure S21**) and the percentage of adaptive walks reaching high peaks increases. Source data are provided with this paper.

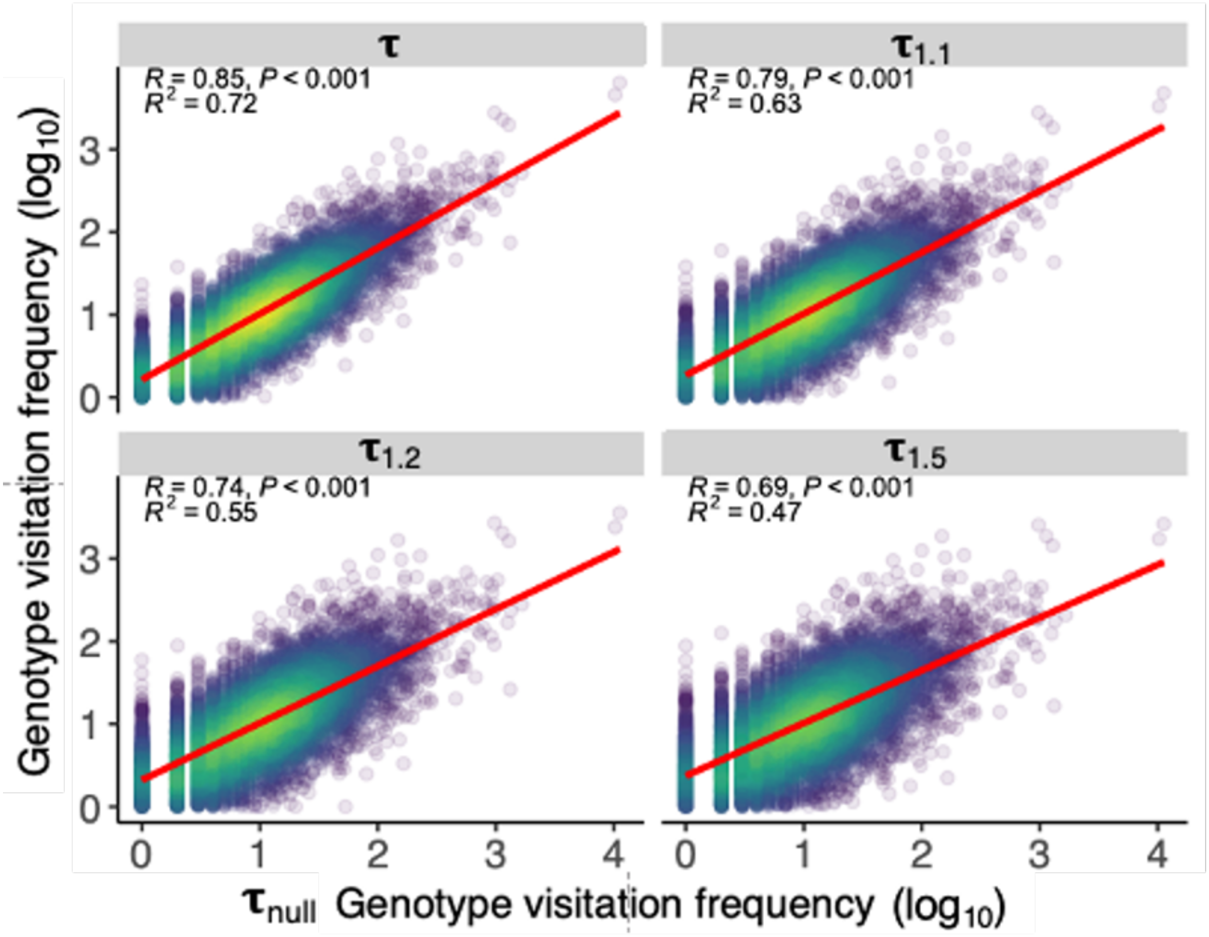

Supplementary Figure S23. Experimental measurement noise has little impact on genotype visitation during adaptive walks. The figure presents a pairwise comparison of the log-transformed number of visits to each genotype in Kimura adaptive random walks starting from 1,000 different genotypes, with 1,000 walks per starting genotype, across distinct experimental noise levels (τ_{null}=0, τ=τ, τ_1=1.1τ, $\tau_{1.2}$= 0.25τ, $\tau_{1.5}$=1.5τ). Each circle represents the visitation frequency of a distinct genotype in all 10^6 adaptive walks, i.e., the number of times each genotype is visited during these walks. Each plot compares the visitation frequency in the absence of experimental noise (x-axis) with the visitation frequency at various noise levels (y-axis, noise level on the top of each panel in grey). The density of genotypes within each plot is indicated by colors (low: purple, high: yellow). The red line in each plot depicts a linear model's fit to the data, illustrating the association between the frequency of visitation of genotypes across distinct noise values. Spearman correlation coefficients (R) are indicated at

the top of each graph, along with the corresponding p-value for testing the null hypothesis that R=0. Source data are provided with this paper.

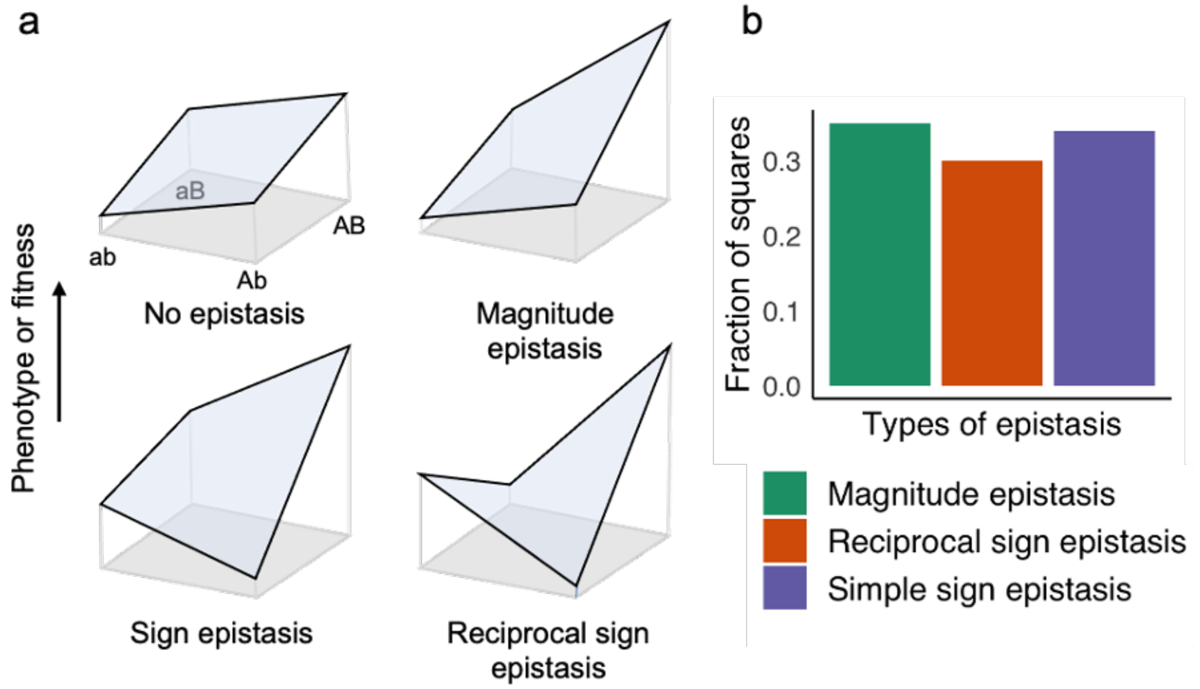

Supplementary Figure S24. Epistatic interactions can influence adaptation and, hence, the ruggedness of adaptive landscapes. a. Overview of epistatic interactions. A ‘wild-type’ sequence (ab) can change to a double mutant (AB) via the single mutations Ab or aB. The upper left panel shows a mutational path without epistasis, where the repression value of the double mutant is the sum of the repression contributions of both single mutants (additive interaction). Magnitude epistasis changes the magnitude, but not the sign of a resulting repression value. In the example, the repression value associated with the AB genotype is higher than the sum of the repression values for Ab and aB (second panel). Sign epistasis occurs when one single mutant (Ab) has a lower repression value than both the ‘wild type’ and the double mutant, while the other single mutant (aB) shows intermediate repression (third panel). In reciprocal sign epistasis, both single mutations decrease repression individually, but increase repression jointly (in the double mutant). Note that the relationship between fitness and repression is dependent on the studied system. In the case of TetR, we have assumed that fitness and repression are positively associated, based on previous studies exploring such relationship[23–26] Figure adapted from[27]. **b. Prevalence of three types of epistasis in the**

landscape. The bar plot shows the prevalence of the three major types of epistatic interactions (panel a) in our data (N=83,100 double mutant pairs). The category called "no sign epistasis" comprises both magnitude epistasis and additivity (no epistasis), without any differentiation between them, as neither of them have an impact on peak accessibility [28,29]. Source data are provided with this paper.

References

1. Aguilar-Rodríguez, J., Payne, J. L. & Wagner, A. A thousand empirical adaptive landscapes and their navigability. *Nat Ecol Evol* **1**, 0045 (2017).
2. Khalid, F. *et al.* Genonets server-a web server for the construction, analysis and visualization of genotype networks. *Nucleic Acids Res* **44**, W70–W76 (2016).
3. Jahn, M., Vorpahl, C., Hübschmann, T., Harms, H. & Müller, S. Copy number variability of expression plasmids determined by cell sorting and droplet digital PCR. *Microb Cell Fact* **15**, 211 (2016).
4. Kelly, J. R. *et al.* Measuring the activity of BioBrick promoters using an in vivo reference standard. *J Biol Eng* **3**, 4 (2009).
5. Sanches-Medeiros, A., Monteiro, L. M. O. & Silva-Rocha, R. Calibrating Transcriptional Activity Using Constitutive Synthetic Promoters in Mutants for Global Regulators in Escherichia coli. *Int J Genomics* **2018**, 1–10 (2018).
6. Pédelacq, J. D., Cabantous, S., Tran, T., Terwilliger, T. C. & Waldo, G. S. Engineering and characterization of a superfolder green fluorescent protein. *Nat Biotechnol* **24**, 79–88 (2006).
7. Hillen, W. & Berens, C. MECHANISMS UNDERLYING EXPRESSION OF TN10 ENCODED TETRACYCLINE RESISTANCE. *https://doi.org/10.1146/annurev.mi.48.100194.002021* **48**, 345–369 (2003).
8. Bertrand, K. P., Postle, K., Wray, L. V. & Reznikoff, W. S. Overlapping divergent promoters control expression of Tn10 tetracycline resistance. *Gene* **23**, 149–156 (1983).
9. Meyer, A. J., Segall-Shapiro, T. H., Glassey, E., Zhang, J. & Voigt, C. A. Escherichia coli "Marionette" strains with 12 highly optimized small-molecule sensors. *Nature Chemical Biology 2018 15:2* **15**, 196–204 (2018).
10. Carr, S. B., Beal, J. & Densmore, D. M. Reducing DNA context dependence in bacterial promoters. *PLoS One* (2017) doi:10.1371/journal.pone.0176013.
11. Lutz, R. & Bujard, H. Independent and tight regulation of transcriptional units in Escherichia coli via the LacR/O, the TetR/O and AraC/I1-I2 regulatory elements. *Nucleic Acids Res* **25**, 1203–10 (1997).
12. Zaslaver, A. *et al.* A comprehensive library of fluorescent transcriptional reporters for Escherichia coli. *Nat Methods* **3**, 623–628 (2006).
13. Lou, C., Stanton, B., Chen, Y. J., Munsky, B. & Voigt, C. A. Ribozyme-based insulator parts buffer synthetic circuits from genetic context. *Nat Biotechnol* (2012) doi:10.1038/nbt.2401.
14. Vlková, M., Morampalli, B. R. & Silander, O. K. Efficiency of the synthetic self-splicing RiboJ ribozyme is robust to cis- and trans-changes in genetic background. *Microbiologyopen* **10**, e1232 (2021).
15. Salis, H. M., Mirsky, E. A. & Voigt, C. A. Automated Design of Synthetic Ribosome Binding Sites to Precisely Control Protein Expression. *Nat Biotechnol* **27**, 946 (2009).
16. Chen, Y. J. *et al.* Characterization of 582 natural and synthetic terminators and quantification of their design constraints. *Nat Methods* (2013) doi:10.1038/nmeth.2515.

17. Bolintineanu, D. S. *et al.* Investigation of changes in tetracycline repressor binding upon mutations in the tetracycline operator. *J Chem Eng Data* **59**, 3167–3176 (2014).
18. Wickham, H. *Ggplot2: Elegant Graphics for Data Analysis*. (Springer-Verlag New York, 2016).
19. Jaccard, P. THE DISTRIBUTION OF THE FLORA IN THE ALPINE ZONE.1. *New Phytologist* **11**, 37–50 (1912).
20. Papkou, A., Garcia-Pastor, L., Escudero, J. A. & Wagner, A. A rugged yet easily navigable fitness landscape of antibiotic resistance. *bioRxiv* 2023.02.27.530293 (2023) doi:10.1101/2023.02.27.530293.
21. Rockel, S., Geertz, M. & Maerkl, S. J. MITOMI: A microfluidic platform for in vitro characterization of transcription factor-DNA interaction. *Methods in Molecular Biology* (2012) doi:10.1007/978-1-61779-292-2_6.
22. Csardi, G. The Igraph Software Package for Complex Network Research. (2014).
23. Berens, C. & Hillen, W. Gene regulation by tetracyclines. Constraints of resistance regulation in bacteria shape TetR for application in eukaryotes. *Eur J Biochem* **270**, 3109–3121 (2003).
24. Nguyen, T. N. M., Phan, Q. G., Duong, L. P., Bertrand, K. P. & Lenski, R. E. Effects of carriage and expression of the Tn10 tetracycline-resistance operon on the fitness of Escherichia coli K12. *Mol Biol Evol* **6**, 213–225 (1989).
25. Eckert, B. & Beck, C. F. Overproduction of transposon Tn10-encoded tetracycline resistance protein results in cell death and loss of membrane potential. *J Bacteriol* **171**, 3557–3559 (1989).
26. Rajer, F. & Sandegren, L. The Role of Antibiotic Resistance Genes in the Fitness Cost of Multiresistance Plasmids. *mBio* **13**, (2022).
27. Poelwijk, F. J., Kiviet, D. J., Weinreich, D. M. & Tans, S. J. Empirical fitness landscapes reveal accessible evolutionary paths. *Nature* (2007) doi:10.1038/nature05451.
28. Weinreich, D. M., Watson, R. A. & Chao, L. Perspective: Sign epistasis and genetic constraint on evolutionary trajectories. *Evolution* (2005).
29. Greene, D. & Crona, K. The Changing Geometry of a Fitness Landscape Along an Adaptive Walk. *PLoS Comput Biol* **10**, e1003520 (2014).
