# Supplementary material for: The highly rugged yet navigable regulatory landscape of the bacterial transcription factor TetR
Source: Nat Commun. 2024 Dec 30;15:10745. doi: 10.1038/s41467-024-54723-y (PMC11686294; doi:10.1038/s41467-024-54723-y)
Supplement: Supplementary file 3 — Reporting Summary [file 41467_2024_54723_MOESM3_ESM.pdf]

Corresponding author(s): Andreas Wagner
Last updated by author(s): 2024.09.04

Reporting Summary

Nature Portfolio wishes to improve the reproducibility of the work that we publish. This form provides structure for consistency and transparency in reporting. For further information on Nature Portfolio policies, see our Editorial Policies and the Editorial Policy Checklist.

Statistics

For all statistical analyses, confirm that the following items are present in the figure legend, table legend, main text, or Methods section.

n/a	Confirmed	
☐	☒	The exact sample size (*n*) for each experimental group/condition, given as a discrete number and unit of measurement
☐	☒	A statement on whether measurements were taken from distinct samples or whether the same sample was measured repeatedly
☐	☒	The statistical test(s) used AND whether they are one- or two-sided<br>*Only common tests should be described solely by name; describe more complex techniques in the Methods section.*
☐	☒	A description of all covariates tested
☐	☒	A description of any assumptions or corrections, such as tests of normality and adjustment for multiple comparisons
☐	☒	A full description of the statistical parameters including central tendency (e.g. means) or other basic estimates (e.g. regression coefficient) AND variation (e.g. standard deviation) or associated estimates of uncertainty (e.g. confidence intervals)
☐	☒	For null hypothesis testing, the test statistic (e.g. *F*, *t*, *r*) with confidence intervals, effect sizes, degrees of freedom and *P* value noted<br>*Give P values as exact values whenever suitable.*
☒	☐	For Bayesian analysis, information on the choice of priors and Markov chain Monte Carlo settings
☒	☐	For hierarchical and complex designs, identification of the appropriate level for tests and full reporting of outcomes
☐	☒	Estimates of effect sizes (e.g. Cohen's *d*, Pearson's *r*), indicating how they were calculated

Our web collection on statistics for biologists contains articles on many of the points above.

Software and code

Policy information about availability of computer code

Data collection

Flow cytometry equipment: BD FACSAria III (BD Biosciences, San Jose, CA)
Flow cytometry software: BD FACSDiva (v9.0)
Plate reader equipment: Tecan Infinite PRO 200
Plate reader software: Tecan i-control software (v 3.14)

Data analysis

Data was analyzed with custom made R and Python scripts that are publicly available on Zenodo: 10.5281/zenodo.8370874
Softwares and packages:
R version 4.1.0 (2021-05-18)
Platform: x86_64-apple-darwin17.0 (64-bit)
Running under: macOS 14.6.1

Matrix products: default
LAPACK: /Library/Frameworks/R.framework/Versions/4.1/Resources/lib/libRlapack.dylib

locale:
[1] en_US.UTF-8/en_US.UTF-8/en_US.UTF-8/C/en_US.UTF-8/en_US.UTF-8

attached base packages:

```
[1] stats4    grid      stats     graphics  grDevices utils     datasets  methods   base

other attached packages:
 [1] tidyr_1.3.1          patchwork_1.2.0      ggpmisc_0.5.5        ggpp_0.5.6           pheatmap_1.0.12
 [6] factoextra_1.0.7     gridGraphics_0.5-1   gplots_3.1.3.1       Biostrings_2.62.0    GenomeInfoDb_1.30.1
[11] XVector_0.34.0       IRanges_2.28.0       S4Vectors_0.32.4     BiocGenerics_0.40.0  ggExtra_0.10.1
[16] ggseqlogo_0.2        scales_1.3.0         cowplot_1.1.3        igraph_2.0.2         vioplot_0.4.0
[21] zoo_1.8-12           sm_2.2-6.0           viridis_0.6.5        viridisLite_0.4.2    cultevo_1.0.2
[26] stringdist_0.9.12    data.table_1.15.0    faux_1.2.1           ggpointdensity_0.1.0 reshape2_1.4.4
[31] GGally_2.2.1         ggridges_0.5.6       ggrepel_0.9.5        ggpubr_0.6.0         ggplot2_3.5.0
[36] dplyr_1.1.4          combinat_0.0-8

loaded via a namespace (and not attached):
 [1] colorspace_2.1-0       ggsignif_0.6.4         ellipsis_0.3.2         rstudioapi_0.15.0      MatrixModels_0.5-3
 [6] fansi_1.0.6            splines_4.1.0          knitr_1.45             polynom_1.4-1          broom_1.0.5
[11] cluster_2.1.6          shiny_1.8.0            compiler_4.1.0         backports_1.4.1        Matrix_1.6-5
[16] fastmap_1.1.1          cli_3.6.2              later_1.3.2            htmltools_0.5.7        quantreg_5.97
[21] tools_4.1.0            gtable_0.3.4           glue_1.7.0             GenomeInfoDbData_1.2.7 Rcpp_1.0.12
[26] carData_3.0-5          vctrs_0.6.5            nlme_3.1-164           xfun_0.42              stringr_1.5.1
[31] mime_0.12              miniUI_0.1.1.1         lifecycle_1.0.4        gtools_3.9.5           rstatix_0.7.2
[36] MASS_7.3-60.0.1        zlibbioc_1.40.0        promises_1.2.1         parallel_4.1.0         SparseM_1.81
[41] RColorBrewer_1.1-3     yaml_2.3.8             gridExtra_2.3          stringi_1.8.3          permute_0.9-7
[46] caTools_1.18.2         rlang_1.1.3            pkgconfig_2.0.3        bitops_1.0-7           evaluate_0.23
[51] lattice_0.22-5         purrr_1.0.2            ggstats_0.5.1          tidyselect_1.2.0       plyr_1.8.9
[56] magrittr_2.0.3         R6_2.5.1               generics_0.1.3         pillar_1.9.0           withr_3.0.0
[61] mgcv_1.9-1             survival_3.5-8         abind_1.4-5            RCurl_1.98-1.14        tibble_3.2.1
[66] crayon_1.5.2           car_3.1-2              KernSmooth_2.23-22     utf8_1.2.4             rmarkdown_2.25
[71] vegan_2.6-4            digest_0.6.34          xtable_1.8-4           httpuv_1.6.14          munsell_0.5.0
```

For manuscripts utilizing custom algorithms or software that are central to the research but not yet described in published literature, software must be made available to editors and reviewers. We strongly encourage code deposition in a community repository (e.g. GitHub). See the Nature Portfolio guidelines for submitting code & software for further information.

Data

Policy information about availability of data

All manuscripts must include a data availability statement. This statement should provide the following information, where applicable:

- Accession codes, unique identifiers, or web links for publicly available datasets
- A description of any restrictions on data availability
- For clinical datasets or third party data, please ensure that the statement adheres to our policy

This paper used experimental data created on its own.
Accession code for Experimental data (Flow cytometry data, plate reader data and files containing the original results from the data analysis): 10.5281/zenodo.8370874 (https://zenodo.org/records/8370874)
Accession code for DNA Sequencing Data: BioSample accessions SAMN37481225, SAMN37481226, SAMN37481227 (https://www.ncbi.nlm.nih.gov/bioproject/PRJNA1019339/)

Research involving human participants, their data, or biological material

Policy information about studies with human participants or human data. See also policy information about sex, gender (identity/presentation), and sexual orientation and race, ethnicity and racism.

Reporting on sex and gender	not applicable
Reporting on race, ethnicity, or other socially relevant groupings	not applicable
Population characteristics	not applicable
Recruitment	not applicable
Ethics oversight	not applicable

Note that full information on the approval of the study protocol must also be provided in the manuscript.

Field-specific reporting

[illegible]

Ecological, evolutionary & environmental sciences study design

All studies must disclose on these points even when the disclosure is negative.

Study description	To assess our plasmid system's ability to capture differences in repression levels of TetR binding site variants, we first measured the GFP expression driven by the wild-type sequence and four previously characterized variants cloned into our plasmid. These measurements showed expression levels consistent with previous reports.<br><br>For subsequent experiments, we used our plasmid system with the wild-type binding site as a positive control for strong transcriptional repression and a negative control without a GFP promoter to set the lower bound of fluorescence as the basal autofluorescence of bacterial cells. We then proceeded to analyze the fluorescence of our library.<br><br>In the absence of the inducer, the wild type promotes strong repression (low fluorescence), whereas the entire TFBS library shows fluorescence that varies broadly. This is the expected behavior if some variants lead to strong repression but others to only weak repression. In the presence of inducer, both the wild type and the entire library experience strong de-represssion (increased fluorescence), which reflects the expected dissociation of TetR from DNA.<br><br>We subdivided the cells from our library into 13 “bins”, based on their observed fluorescence values and sequenced variants from each. We performed this sorting procedure in triplicates with three independent cell transformations. The resulting library contained 17,851 genotypes, which represent 27% of the 48 genotypes in the genotype space we study. Although we did not recover all genotypes, the correlation of read counts for each variant was remarkably consistent across replicates. The Pearson's correlation coefficient R for replicates ranged from 0.971 to 0.991. We removed reads sequences with low read coverage. We used the observed distribution of individual variants among “bins” to map genotypes to their respective repression levels. We normalized repression levels by the wild type, such that values below one indicated weaker repression than the wild type, while values above one indicated stronger repression. To validate the data from our sort-seq experiments, we compared fluorescence levels from a plate reader assay with our sort-seq calculated repression levels for 15 variants from each bin, i.e., for a total of 13x15=195 variants. The correlation between the two measurements was strong and nearly linear (Pearson R=-0.86, p<0.001, N=195).
Research sample	We used Escherichia coli SIG10 MAX Electrocompetent Cells (Sigma Aldrich) for clononing and sort-seq experiments<br>The strain genotype is similar to DH5Alpha (F- mcrA Δ(mrr-hsdRMS-mcrBC) endA1 recA1 Φ80dlacZΔM15 ΔlacX74 araD139 Δ(ara,leu) 7697galU galK rpsL nupG λ- tonA (StrR))
Sampling strategy	Sampling of colonies from each bin for for plate reader experiments was at random.<br>For the simulations there was no random sampling for determining starting points. All non-peak nodes were used.<br>For adaptive walks using an uniform branching approach, every time immediate neighbors from a sequence presented higher repression values. One of them was selected at random for proceeding the walk.
Data collection	Flow cytometry data was collected by the first author on a BD FACSAria III, (BD Biosciences, San Jose, CA). A 70 µm nozzle (70 psi sheath pressure, 89 kHz drop drive frequency, 5-6 gap value) was used. Prior to sorting, AccuDrop beads were used to calibrate the drop delay (specific to each experiment, but usually between 46 and 48). The lowest possible threshold for particle detection was set (FSC & SSC > 200). Fluorescence (FITC-H channel, 488nm laser, emission filters 502LP, 530/30). Voltages: FSC: 625V; SSC: 420V; FIT-C:600V. Sorted cells were collected in 1.5 mL Eppendorf tubes with LB antibiotic-free media maintained 4°C. Data was recorded on a BD FACSDiva v9.0 Software. The full SOP can be found here: https://www.cytometry.uzh.ch/dam/jcr:4c573ae2-31c1-411f-82ab-a1713f8104a2/Aria_Irchel_1_SOP_2021-05-11.pdf
Timing and spatial scale	Cell cultures were consistently collected during the late exponential or early stationary phases to capture cells at a point of high metabolic activity. Cell sorting was conducted over four consecutive days to maintain culture freshness and minimize any freeze/thaw effects on cell physiology and gene expression. The protocol timing was as follows:<br>Day 1: Cells were revived from a frozen library aliquot by inoculating 50 µL into 5 mL of LB-Cm and grown overnight.<br>Day 2: 50 µL of the initial culture was transferred to fresh LB-Cm with Atc, followed by overnight growth.<br>Day 3: Cells were sorted, and 50 µL of the sorted culture was inoculated again in LB-Cm with Atc for overnight growth to ensure recovery.<br>Day 4: A second round of cell sorting was performed on the recovered culture.<br>Day 5: Glycerol stocks were prepared, and plasmids were extracted using miniprep for sequencing preparation.<br>Day 6: PCR amplification of the transcription factor binding site (TFBS) region was conducted, followed by purification and sequencing.
Data exclusions	For the sequencing analysis, sequences with less than 30 read counts (paired) and a coefficient of variation (based on triplicates) higher than 50% were excluded.
Reproducibility	Each plate reader assay was performed in triplicates (three biological replicates per sample with three technical replicates per biological replicate).<br>Each sorting procedure was performed in triplicates by three independent transformations followed by sorting and sequencing of bins.
Randomization	Not applicable
Blinding	Not applicable. Blinding was not applicable to our study as the properties we were assessing were only apparent after the measurements were made. In other words, the experimenter is blind to the results until the data analysis is completed.

Did the study involve field work? ☐ Yes ☒ No

Reporting for specific materials, systems and methods

We require information from authors about some types of materials, experimental systems and methods used in many studies. Here, indicate whether each material, system or method listed is relevant to your study. If you are not sure if a list item applies to your research, read the appropriate section before selecting a response.

Materials & experimental systems

n/a	Involved in the study
☒	☐ Antibodies
☒	☐ Eukaryotic cell lines
☒	☐ Palaeontology and archaeology
☒	☐ Animals and other organisms
☒	☐ Clinical data
☒	☐ Dual use research of concern
☒	☐ Plants

Methods

n/a	Involved in the study
☒	☐ ChIP-seq
☐	☒ Flow cytometry
☒	☐ MRI-based neuroimaging

Flow Cytometry

Plots

Confirm that:

☒ The axis labels state the marker and fluorochrome used (e.g. CD4-FITC).

☒ The axis scales are clearly visible. Include numbers along axes only for bottom left plot of group (a 'group' is an analysis of identical markers).

☒ All plots are contour plots with outliers or pseudocolor plots.

☒ A numerical value for number of cells or percentage (with statistics) is provided.

Methodology

Sample preparation	We grew populations of E. coli cells hosting the library in LB medium supplemented with 50 µg/mL chloramphenicol to saturation (overnight growth, 16 hours, 200 rpm, 37°C). After overnight growth, we diluted the overnight cultures in LB medium supplemented with 50 µg/mL chloramphenicol in a 1:100 ratio (v/v) and grew the cultures for 5 h until they reached late-exponential/early -stationary phase. Before sorting, we diluted 50µL of the cultures in 1mL of cold Dulbecco's PBS (Sigma-Aldrich #D8537) in 15 mL FACS tubes.
Instrument	BD FACSAria III, (BD Biosciences, San Jose, CA)
Software	BD FACSDiva v9.0 Software and RStudio for analysis
Cell population abundance	10^6 sorted cells per sorting
Gating strategy	We chose the FITC channel voltage such that the median fluorescence of the negative control sample (promoterless pCAW-Sort-Seq plasmid, without sfGFP expression) was between 0 and 100 (arbitrary units) on the FITC-H axis.<br>We set sorting gates on the FITC-H axis as follows: First, we recorded autofluorescence of the negative control cell culture. The median autofluorescence from this control served as the upper boundary of the lowest bin (B1) for the experimental population. Then we recorded the fluorescence of 10^6 cells expressing sfGFP and harboring the library, without sorting. The choice of this number (10^6) of cells was based on the combination of library size and the estimated loss of library diversity post-sorting (a reduction of up to 70% in sequence diversity was found in similar sort-seq studies). Thus, we estimated that 975,000 cells would be required to represent the whole library (N= 65,536) with at least 5 cells harboring a copy of each sequence and accounting for diversity loss. We then proceeded to set our binning gates. We took the lower bound of the highest bin (B13) for the experimental population to correspond to the 95th percentile of the fluorescence distribution of this population. We chose boundaries between intermediate bins with equidistant spacing on a binary logarithmic (log2) scale. After we had the gates in this way, we calculated the fractions of the previously 10^6 recorded cells that were inside each gate.<br><br>We determined the number of cells to be sorted into each of the 13 bins from the fraction of cells we had previously recorded in each of the bins, such that the total number of sorted cells was equal to 106. We sorted cells into 1.5 mL Eppendorf tubes with 500 µL of LB medium each. We kept the tubes cooled to 4 °C to halt growth while sorting, and during the sorting of subsequent samples. We carried out the sorting procedure in three replicates derived from three independent library transformations.

☒ Tick this box to confirm that a figure exemplifying the gating strategy is provided in the Supplementary Information.
